# Risk Assessment of Displaced Sediment by an Extreme Event Cyclone in a Peri-Urban Zone Using Bioassays and Analytical Chemistry

**DOI:** 10.3390/toxics12080558

**Published:** 2024-07-31

**Authors:** Louis A. Tremblay, Daisuke Nakajima, Satoshi Endo, Mayuko Yagishita, Hannah Ludlow, Ariana Mackay, Olivier Champeau

**Affiliations:** 1 Cawthron Institute, Private Bag 2, Nelson 7042, New Zealand; olivier.champeau@epa.govt.nz; 2 School of Biological Sciences, University of Auckland, Auckland 1142, New Zealand; 3 Manaaki Whenua–Landcare Research, Lincoln 7608, New Zealand; 4 Health and Environmental Risk Division, National Institute for Environmental Studies, Tsukuba 305-8506, Japan; dnakaji@nies.go.jp (D.N.); endo.satoshi@nies.go.jp (S.E.); 5 Department of Life and Environmental Science, Prefectural University of Hiroshima, Hiroshima 734-8558, Japan; yagishita@pu-hiroshima.ac.jp; 6 Hawke’s Bay Regional Council, 159 Dalton Street, Napier 4110, New Zealand; hannah.ludlow@pdp.co.nz (H.L.); ariana.mackay@hbrc.govt.nz (A.M.); 7 Pattle Delamore Partners, Ground Floor South, Bower House, 18 Bower Street, Napier 4110, New Zealand

**Keywords:** New Zealand, Microtox, mussel embryo test, yeast two-hybrid assay, aryl hydrocarbon receptor, constitutive androstane receptor, AIQS-GC, DDT, phytosterol

## Abstract

Hawke’s Bay in New Zealand was impacted by Cyclone Gabrielle in 2023, experiencing intense weather conditions and rainfall. Rivers and streams surged beyond their banks, displacing large amounts of sediment. The sewage treatment plant and industries in the Waitangi catchment, south of the city of Napier, were heavily impacted, making them potential sources of contaminants. The aim of this study was to investigate the risk of displaced sediments deposited south of Napier City, using bioassays and chemical analysis methods. Sediment samples were collected across a gradient between the coastline and the Waitangi Stream. The toxicity of chemically extracted or elutriate samples was assessed by Microtox^®^, mussel embryo–larval development, and aryl hydrocarbon and constitutive androstane receptor yeast two-hybrid assays. Targeted chemical analysis and automated identification and quantification system (AIQS-GC) methods were used to identify contaminants. The elutriates showed low toxicity and the yeast assays showed levels of activity like those previously reported. Chemical methods confirmed historical contamination by DDT and its metabolites DDE and DDD, as well as by plant sterols. Overall, the toxicity and chemicals detected are what would be expected from a typical agricultural soil. The risk posed by the displaced sediment in the Waitangi catchment can be considered low. Combining chemical and bioanalytical methods was an effective approach to investigate the potential risks of post-disaster contamination.

## 1. Introduction

Studies on the effect of climate change on weather patterns are strongly in agreement that the frequency of atmospheric rivers will increase globally [1]. Atmospheric rivers are defined as “long, narrow, and transient corridor[s] of strong horizontal water vapor transport that is typically associated with a low-level jet stream ahead of the cold front of an extratropical cyclone” [2]. Because of its insularity and sharp topography almost perpendicular to the dominant westerly winds at latitudes where oceans largely prevail, New Zealand is recurrently affected by atmospheric river events [2].

On 14 February 2023, ex-tropical cyclone Gabrielle impacted several countries [3]. Gabrielle was a category 3 tropical cyclone and is precisely the type of system that society would like to have a better understanding of in a changing climate [3]. The maximum rainfall recorded in the New Zealand North Island Hawke’s Bay region by Hawke’s Bay Regional Council was 501 mm in 24 h. The cyclone generated strong winds resulting in widespread damage and flooding. Rivers burst their banks, flooding homes and businesses. The Awatoto suburb south of Napier City was completely inundated, including the wastewater treatment plant, the surrounding industrial area [4], and the Waitangi Stream catchment, potentially spreading contaminants across the land. There were concerns over the potential risks posed by the silt and sediment that were displaced and covered the Waitangi Stream during the cyclone event.

Floods are the most common type of disaster globally that have been associated with multiple deaths [5]. Intense weather events can potentially displace chemical contaminants depending on the locations mostly affected. Flooding can result in the accumulation of trace metals at concentrations higher than health criteria, as reported in sediment samples from Texas following Hurricane Harvey [6]. Organic contaminants can also be displaced. A study on the impacts of Hurricane Katrina in the New Orleans area detected mixtures of chemicals at many sampling locations following the event [7]. There is indication that the level and type of contaminants displaced is correlated with the presence of contaminated sites. This was suggested in an investigation of the concentrations of a range of contaminants in soil samples collected from the metropolitan New York City following Hurricane Sandy [8].

The aim of this study was to assess the toxicity of sediment samples collected from the Waitangi Stream to provide an indication of chemical contamination using chemical analysis and bioassays. For bioassays, we used bacteria and mussel embryo development standard tests to assess the general toxicity of the sediment samples for contaminants likely associated with urban areas. Two yeast-based assays were used to determine the presence of chemicals commonly found in urban environments based on their biological activities. The aryl hydrocarbon receptor (AhR) and the constitutive androstane receptor (CAR) are ligand-activated transcription factors involved in xenobiotic metabolism. Dioxin-like compounds are the ligands of the AhR and include molecules such as polychlorinated dibenzo-p-dioxins (PCDDs), polychlorinated dibenzofurans (PCDFs), polychlorinated biphenyls (PCBs), and polycyclic aromatic hydrocarbons (PAHs). CAR has a low specificity for ligands and is implicated in hepatic detoxification of endogenous molecules, xenobiotics, and environmental contaminants. Both AhR and CAR yeast assays have detected these biological activities for many chemicals [9,10]. The presence of chemical contamination was characterised using the combination of a rapid comprehensive chemical method with a GC–MS equipped with an automated identification and quantification system (AIQS-GC) and targeted chemical analyses [11].

## 2. Materials and Methods

### 2.1. Reagents

Solvents were of the grade used to test pesticide residue and polychlorinated biphenyl (Nacalai tesque and FUJIFILM Wako Pure Chemical Corporation, Osaka, Japan). The NAGINATA internal standard Mix II (500 μg/mL in acetone; 4-chlorotoluene-d4, 1,4-dichlorobenzene-d4, naphthalene-d8, acenaphthene-d10, phenanthrene-d10, fluoranthene-d10, chrysened12, and perylene-d12) and NAGINATA criteria sample Mix II (in dichloromethane) were from Hayashi Pure Chemical Ind., Ltd. (Osaka, Japan).

### 2.2. Sample Collection and Pretreatment

**Figure 1:**
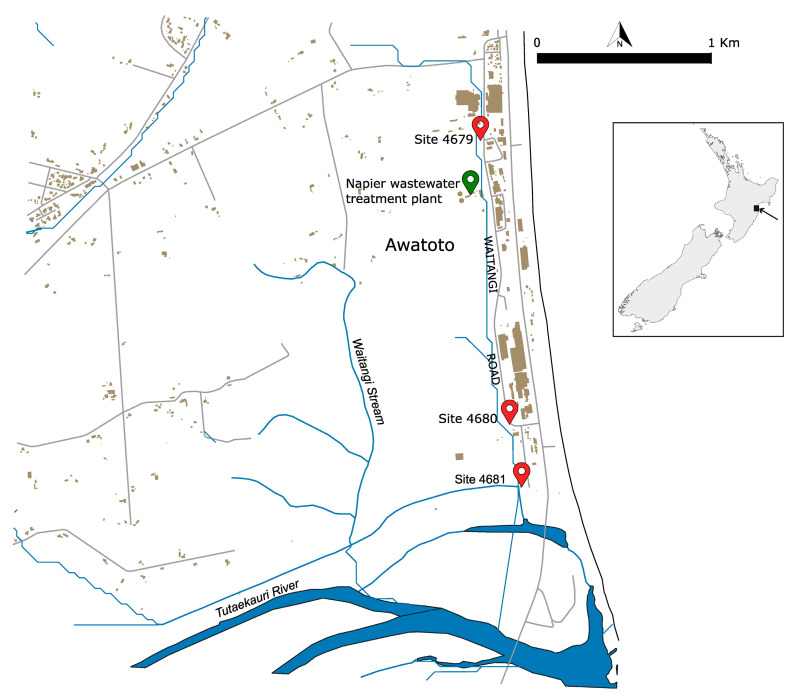
Location of the sediment sampling sites in the Awatoto suburb south of Napier City in Hawke’s Bay, located on the east coast of the North Island of New Zealand, as per the insert on the right. The sites are indicated by the red symbols along with their respective identification numbers. The green symbol indicates the location of the Napier sewage treatment plant.

Sediment samples were collected by the Hawke’s Bay Regional Council staff on 13 May 2023 at three locations (Figure 1):

- Waitangi Stream opposite 32 Waitangi Road (Site 4679) (latitude −39.54070, longitude 176.91752);
- Waitangi Stream D/S BioRich driveway (Site 4680) (latitude −39.55579, longitude 176.92030);
- Waitangi Stream D/S stopbank—receiving environment (Site 4681) (latitude 39.55939, longitude 176.92128).

A stainless-steel trowel was used to scrape approximately the top 20 mm of deposited sediment from 10 to 12 repeats along a 5 m stretch running parallel to the direction of the flow in the deepest part of the channel for an approximate total of 2000 g wet weight of sediment. This sediment was deposited in a plastic zip bag for homogenisation, after which a portion was transferred to two polyethylene bottles and sent to Hill Laboratories Ltd. for a range of organic chemical analyses. The remaining sediment in the zip bag was sealed, double-bagged, and sent to the Cawthron Institute for toxicity testing, as well as preparation and shipping to the National Institute for Environmental Studies (NIES), Japan for additional bioassay testing and AIQS-GC analyses.

### 2.3. Toxicity Testing

#### 2.3.1. Elutriates and Sediment Extract Preparations

Sediment samples were mixed with the dilution water corresponding to each test at a ratio of 1:4 sediment/dilution water [12]. Two water types—including a solution of 2% sodium chloride (NaCl) filtered at 0.22 μm and reconstituted seawater filtered at 0.45 μm—were used to dilute the test solutions for the Microtox^®^ and the blue mussel embryo–larval tests, respectively.

The mixture of sediment/diluent was mixed in a rotating block for 1 h in the dark at 4 °C. It was then centrifuged for 2 × 3 min at 1500× *g* to achieve a clear supernatant without any coloration or obvious suspended particles. The alternative method of letting sediment settle to achieve a clear supernatant took too long (>1 h), likely due to the fine silt.

Supernatants were then used immediately to generate a dilution series for the assays with their respective diluents. Tests were carried out within 1–4 h after elutriates were prepared. Elutriates were diluted two-fold to achieve the range of concentrations for testing.

For the non-targeted chemical analysis, 20 g of sediment (wet weight) sample was first extracted with 20 mL of acetone by shaking and sonication for 10 min. It was further extracted with a 20 mL solution of acetone and dichloromethane (DCM) (1:1) using the same method, and the resulting extracts were then combined (40 mL in total). Of the total 40 mL, a volume of 20 mL consisting of 5 mL of DCM and 15 mL of acetone was used as the analysis sample. Then, 100 mL of pure water and 3 g of NaCl were added to a 20 mL volume, and 5 mL of DCM was recovered after shaking at 270 rpm for 10 min. The DCM was dried under a nitrogen stream and redissolved in 1 mL n-hexane. The internal standard (10 μL) for NAGINATA was added to a 100 μL sample to prepare a sample for AIQS-GC analysis [11]. NAGINATA is a software of AIQS-GC (Nishikawa Keisoku Co., Ltd., Shibuya, Kanagawa, Japan) that can identify and quantify chemicals using AIQS-database.

In parallel, the moisture content of the sediment samples was determined following a recognised standard protocol [13], where a known weight of sediment was dried at 105 °C until a constant weight was reached to estimate the dry weight.

#### 2.3.2. Bioassays

The potential sediment toxicity was assessed using a combination of in vitro and whole organism assays. The in vitro assays provide insights into the presence and bioactivity of chemical contamination translocated in the soil during a flood event, while the whole organism assays provide a general toxicity indication of the contaminants from the sediments that could be resuspended in water.

- In vitro yeast two-hybrid assay

Yeast two-hybrid (Y2 H) assays with yeast cells (*Saccharomyces cerevisiae* Y190) incorporating human CAR and AhR have been previously developed and described [9,14]. CAR and AhR are ligand-activated transcription factors involved in xenobiotic metabolism. Y2 H is a genetically modified assay incorporating the chemiluminescent reporter gene (for β-galactosidase) as a marker of activity in 96-well culture plates.

Yeast cells for the CAR binding assay were pre-incubated at 30 °C for 24 h while shaking in the medium (Table S1), and the cell density was adjusted to an absorbance of 1.75–1.85 at 595 nm. The medium (60 μL) was added to each well of the first row of a black 96-well culture plate for chemiluminescence measurement.

The medium (60 μL) was added to the wells in all cells. Sediment extracts (in DMSO, 20 μL) were added to the medium (480 μL), and aliquots of this mixture (60 μL) were also added to the wells of the first row of the plate. The test solution was serially diluted from rows 1 to 7 (each 2), then the yeast cell suspension (60 μL) was added to each well (including those in row 8, which served as the blank control).

After adding the yeast suspension and vortexing, the plates were incubated at 30 °C under high humidity for 4 h. Next, 50 μL of lysis solution (zymolyase 100 T/Z buffer [2.9 mg/10 mL]) was added to each well, mixed, and left at 37 °C for 1 h. The solution (80 μL) for inducing chemiluminescence from released β-galactosidase, consisting of a reaction buffer (Table S2) containing enhancer (Sapphire-II, Applied Biosystems, Tokyo, Japan) and substrate (Galacton-Star, Applied Biosystems, Tokyo, Japan), was added to each well. The plate was incubated on a hotplate set at 30 °C for 10 min and then placed in a 96-well plate luminometer (Luminescencer JNRAB2100, Atto Corporation, Tokyo, Japan). Agonist activity was evaluated as ECx10, defined as the concentration of test solution producing a chemiluminescent signal 10 times that of the solvent control. 4-tertoctyl-phenol (4 op) was used in the positive control of the Y2 H for CAR. The sample’s activity was calculated by converting it into each positive control equivalent concentration, compared with the 4 op activity on that day. The activity was calculated as wet weight (ww) and converted to dry weight (dw) using the moisture content of each sediment sample.

Yeast cells for AhR were pre-incubated with shaking for 24 h at 30 °C in medium (Table S1) supplemented at 1% with filter-sterilised leucine aqueous solution (1.76 mg/100 mL).

After measuring the cell density of the bacteria at 595 nm, the yeast suspension was centrifuged to precipitate it and the supernatant was decanted. Cell density was adjusted to an absorbance of 1.75–1.85 using the reaction solution medium (Table S2).

The next stage in the procedure was similar to that applied for CAR. The final composition of the medium for the assay is reported in Table S3. AhR activity values were calculated using β-naphthoflavone (βna) as a positive control.

- Microtox^®^ (*Aliivibrio fischeri*) bioluminescence inhibition

Microtox^®^ determines the acute toxicity of aqueous solutions by measuring the changes in light naturally produced by the bioluminescent bacterium *Aliivibrio fischeri* under standard conditions when exposed to a test sample [15]. The test was conducted according to the relevant International Organization for Standardization standard [16]. In brief, freeze-dried bacteria in a vial were revived in a 5 °C aqueous suspension and added at 1% in the test solution. The luminescence in test samples was measured after 5, 15, and 30 min at 15 °C, with the measured effect, light reduction, relating to the control. A summary of the test conditions is presented in Table S4.

- Blue mussel (Mytilus galloprovincialis) embryo–larval development/survival

Adult blue mussels were collected from Pelorus Sound/Te Hoiere in early winter (sea-water temperature: 13.5 °C, salinity: 34.9 psu, oxygen saturation: 85.4%, and pH: 8.09). The animals were kept in a recirculatory system at 13 °C until use. The test was conducted according to the ASTM standard [17]. A summary of the test conditions is presented in Table S4.

The survival of mussel larvae was determined by the yield of normal D-larvae characterised under the microscope. The number of abnormal D-larvae in a test solution provides an indication of embryo toxicity in early life stage development. Survival at each concentration is compared to survival in the control to assess the ecotoxicological parameters (10%, 25%, and median lethal concentration).

### 2.4. Chemical Analysis

#### 2.4.1. Rapid Comprehensive Chemical Analysis

Analyses were conducted on Agilent 5977 B MSD (measurement parameters provided in Table S5). Sediment extracts from each sediment sample were analysed using AIQS-GC methods, as previously described, including validation processes [11,18]. Information on retention times (RTs), retention indexes (RIs), mass spectra, and calibration curves were registered in the AIQS database (AIQS-DB). A chemical was identified by comparing RTs or RIs and the mass spectrum in the sample with the AIQS-DB. The chemical analyses presented here used AIQS-GC for the identification of each chemical in the environmental sample.

Chemicals identified in the AIQS-GC were allocated between one and five stars based on the identification accuracy using the star-based scoring conditions presented in Table 1. Target concentrations below the method of detection limits were treated as zero. A mass spectrum for nonylphenol is provided as an example (Figure S1).

#### 2.4.2. Targeted Chemical Analysis

Sediment samples collected were sent to Hills Laboratory Ltd. for analysis for a suite of metals and metalloids, and an extensive suite of organic chemicals (heavy metals, pesticides, polychlorobiphenyls, ethers, hydrocarbons (polycyclic aromatic and total petroleum), phenols, plasticisers, and halogenated compounds. Details are provided in Table S6. For the organic chemicals, the results of the measured concentrations in the sediment samples were normalised to 1% total organic carbon to allow comparison with the available default guideline values [19].

### 2.5. Statistical Analysis

A Shapiro–Wilk test and a Levene test were used to check the normality of data distribution and homogeneity of variances, respectively. When data did not follow a normal distribution, an arcsine transformation was carried out. An ANOVA was conducted to determine the significance of effects (*p* < 0.05), followed by a Dunnett test as a post hoc analysis. Model-based ecotoxicological parameters (LC_10_ and LC_50_ with associated 95% confidence intervals (CI), and no effect (significant) concentration (N(S)EC) were calculated using R [20] with the drc [21] and bayesnec [22] packages, respectively. A post hoc Dunnett test was used for comparison with the control treatment. Hypothesis testing (no effect and lowest effect observed concentrations (NOEC and LOEC) at the level of statistical significance of *p* < 0.05) were determined with Statistica 14.0 software (TIBCO Software Inc., Palo Alto, CA, USA, 2020).

## 3. Results

The sediment samples were homogenous in colour and texture. They can be classified as ‘mud’ (mix of silt and clay, with a majority sediment grain size <63 µm (Table S6). No dark colour, which is typically characteristic of anoxic conditions, was observed. The moisture content of the three sediment samples used for bioassays was 47%, 43%, and 47% for 4679, 4680, and 4681, respectively (similar to the dry matter reported in Table S6).

### 3.1. Bioassays

#### 3.1.1. AhR and CAR

The results from the AhR and CAR binding activity using Y2 H reporter gene assays are summarised in Table 2. AhR- and CAR-mediated activities were detected in all sediment sample extracts tested. The AhR activities were higher than those reported for sediment samples displaced by floods caused by Typhoon Hagibis in Japan in October 2019 [11]. The CAR activities detected were also higher than those reported for the sediment samples from Typhoon Hagibis, indicating the presence of 4-tertoctyl-phenol-type substances.

#### 3.1.2. Microtox^®^

**Figure 2:**
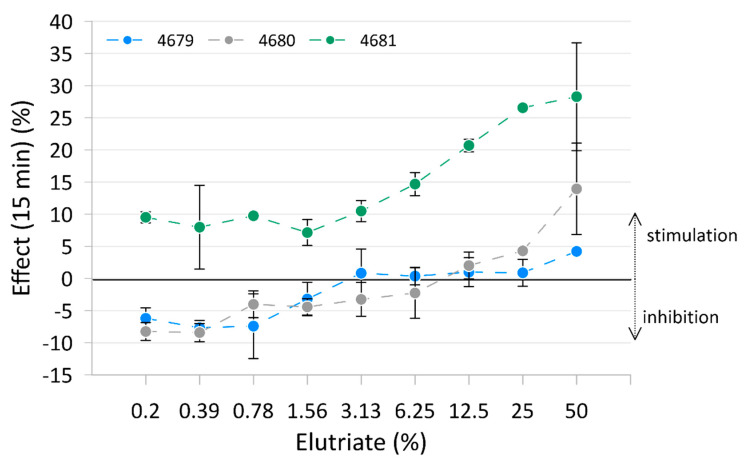
Mean effect (± standard deviation) on bacterial luminescence of the three tested elutriates.

The results of the Microtox^®^ test are presented in Figure 2, with raw data reported in Table S6 and Figure S2. Only measurements after 15 min of exposure are shown in the figure as these best represent the trend. Measurements after 5 min and 30 min are reported in Table S7. Elutriates from sediment samples from Site 4679 and Site 4680 inhibited light emission in bacteria at the lowest concentration but stimulated it at the highest concentration. Elutriate from sediment samples from Site 4681 showed the stimulation of light emission from the bacteria at all concentrations. No significant toxicity of the elutriates measured as bioluminescence inhibition was detected.

#### 3.1.3. Blue Mussel Embryo–Larval Development Assay

The conditions of the assays (temperature, salinity, oxygen saturation, dissolved oxygen concentration [DO], and pH) were measured at the highest elutriate concentrations and are presented in Table S8. The survival of larvae in the controls was below the required 60% and, therefore, did not comply with test validity requirements. The sensitivity of the test, assessed with the response to the reference toxicant (0.148 (0.144–0.153) mg Zn^2+^/L), was within the required limits of ±2 standard deviations of historical data ([0.115–0.233] mg Zn^2+^/L, *n* = 8). In this case, the ecotoxicity parameters can be misestimated, but they can still allow comparisons between the effects of the elutriates.

**Figure 3:**
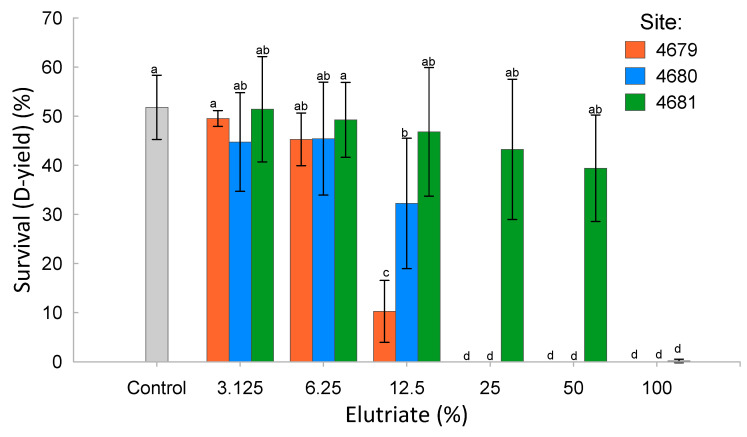
Mean and standard deviation of the blue mussel larvae survival over a range of concentrations of elutriates from the three sediment samples (a same letter indicates a statistical non-significance, *p* < 0.05).

Blue mussel larvae survival in relation to elutriate concentration is reported in Figure 3. Ecotoxicity parameters are reported in Table 3. The ecotoxicity parameters associated with sediment elutriate from Site 4679 were the lowest compared to the two other sites, indicating the strongest toxic effects on mussel larvae development. The least toxic sediment elutriate was from Site 4681, which showed the lowest impact on mussel larvae development. The sediment elutriates from Site 4680 had an intermediate impact. The raw data for the embryo–larval development assay are reported in Table S9.

### 3.2. Chemical Analysis

#### 3.2.1. Rapid Comprehensive Chemical Analysis

**Figure 4:**
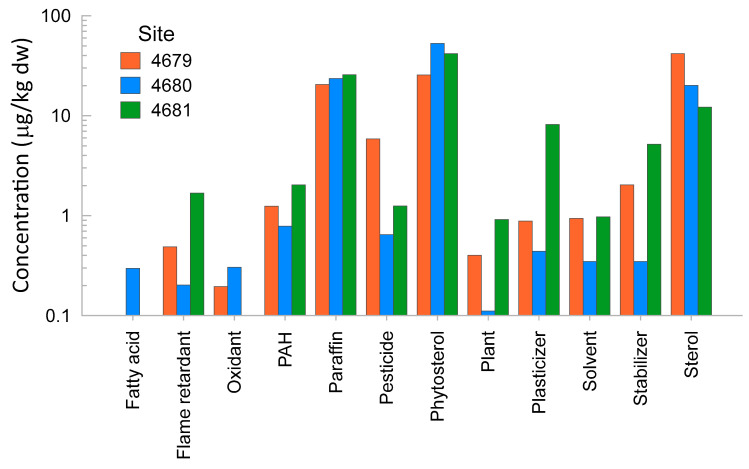
Main categories of chemicals detected and their relative abundances in the three sediment extract samples.

The numbers of compounds detected in sediment extracts were 52, 46, and 55 for the samples from Sites 4679, 4680, and 4681, respectively. Details of the detected compounds are presented in Table S10. The most abundant chemicals found in the three sediment extracts were phytosterols, sterols, and ‘paraffins’ (Figure 4). The sterols found were cholesterol and its metabolites. ‘Paraffins’ are long carbon chains and can be found in oil, lubricants, fuel, and plant products. The extract from Site 4679 showed the highest concentration of pesticides, or chemicals used in the manufacture/formulation of pesticides and sterols. The extract from Site 4680 showed the highest concentration of phytosterol. The extract from Site 4681 had the highest concentration of flame retardants, PAHs, plasticisers, stabilisers, and plant products (Figure 4). The rapid comprehensive chemical analysis detected the presence of DDT metabolites at the highest concentrations in Site 4681, followed by Site 4680. The lowest concentrations were found in the Site 4679 sample extract.

#### 3.2.2. Targeted Analysis

Full details of the measured and detected compounds and their concentrations are presented in Table S6. Metal and metalloid concentrations in sediments were all below the Australia and New Zealand default guideline values (DGVs) for sediment [19] (Table 4). The total organic carbon content in sediments was 1.82%, 1.45%, and 2.4% for the samples from Sites 4679, 4680, and 4681, respectively. A limited number of organic chemicals (DDT and its metabolites, bifenthrin, and dieldrin) were detected. Their concentrations were normalised to a carbon content of sample of 1% for comparison against the DGVs. The chemicals and their concentrations are reported in Table 5, along with the related DGVs. Site 4680 had the highest concentration of DDT metabolites (DDD and DDE) above the DGV high. Site 4679 had the lowest concentration of DDT and metabolites (only the DDE concentration was above DGV). Site 4680 was intermediate. Bifenthrin was detected above the Environmental Quality Standards for Priority Substances under the European Water Framework Directive [23] (no Australia and New Zealand DGV available) for Site 4679 and, at a lesser concentration, at Site 4681.

## 4. Discussion

In recent years, there has been an increase in the number of severe weather events globally that can result in the remobilisation of pollutants [24]. Flood events can redistribute sediment-bound chemicals, and robust risk assessment processes are required to inform communities and develop effective remediation management actions. The approach of this study to characterise the risk of the relocated sediment in the Waitangi catchment has been used successfully to assess the risk of the aftermath of Typhoon Hagibis in Japan [11]. The combination of bioassays and chemical analysis can characterise the toxicity and identify the likely chemicals responsible for the biological activities.

The Microtox^®^ and mussel embryo–larval development assays provide insights into the general toxicity of a sample. The AhR and CAR binding assays provide more specific information about the presence of chemicals with specific mechanisms of toxicity through binding to these receptors. The Microtox^®^ results suggest low overall toxicity in the elutriates from the three sediment samples. Elutriates from sediments from Site 4679 and Site 4680 were slightly toxic, while those from Site 4681 stimulated bacterial growth and, therefore, may have contained nutrients beneficial to *Aliivibrio fischeri*. It has been reported that some substances can stimulate the growth of bacteria and modulate the response to toxicants [25,26]. Cytotoxicity was detected in pre- and post-hurricane season samples in the Houston area and the authors suggested that higher concentrations of metals and nutrients might be contributors to significant cell toxicity [27]. The results of the mussel embryo–larval development assay agreed with the Microtox^®^ results, with the elutriate from the Site 4681 sediment sample being the least toxic, followed by Site 4680 and with Site 4679 being the most toxic. It should be noted that pH values were lower at higher elutriate concentrations, which could be a confounding factor for the toxicity observed in the embryo–larval test. The sediment elutriate dilutions for which the pH fell below 7 were 25%, 50%, and 100% for the elutriates from Site 4679, 4680, and 4681 sediment samples, respectively. Embryo–larval bioassays are used to assess sediment toxicity (e.g., [28]).

In vitro bioassays have been used to assess the toxicity of sediment samples as part of effect-based monitoring [29]. The AhR- and CAR-mediated activities were the highest in the sediment sample from Site 4681, followed by that from Site 4679. This finding was opposite to the results from the Microtox^®^ and mussel embryo–larval development assays, where the Site 4681 sample had the lowest toxicity. It is important to note that the yeast tests were carried out on chemically extracted sediment samples, which means that they contained hydrophobic chemicals that are likely absent from the elutriate samples. As such, they complement the results obtained from the tested elutriates, which provide insights into the more water-soluble chemicals present in the sediment. The results mean that there are more toxic hydrophobic chemicals that can bind to the AhR receptor in samples from Sites 4679 and 4681 compared to the Site 4680 sample.

The chemical analysis detected low DDT concentrations in the sediment samples, but some of its metabolites were high (above DGVs; Table 5). This suggests historical contamination, where legacy DDT has degraded. It has been reported that agricultural soils in Aotearoa New Zealand where DDT was applied to pasture contain persistent residues [30]. The half-life of DDT in soil is 2–10 years, and DDD (dichlorodiphenyldichloroethane) and DDE (dichlorodiphenyldichloroethylene) are the two main metabolites resulting from its degradation [31]. The degradation products DDE and DDD are less toxic than DDT but remain persistent in the environment [32,33].

Bifenthrin is the other pesticide that was measured in the sediment samples at concentrations above its DGV (Table 5). Bifenthrin is a pyrethroid insecticide that affects the nervous system of the insects it targets. It has a broad range of activities used against foliar pests, including Coleoptera, Diptera, Heteroptera, Lepidoptera, Homoptera, and Orthoptera, among others. Bifenthrin is highly acutely toxic to aquatic species and its metabolites can cause endocrine disruption and immunotoxicity [34]. It is allowed for use, with controls, in Aotearoa New Zealand in various formulations to control insects in commercial and residential applications. It has a moderate to high persistence in soil, with a half-life of 7 days to 8 months [35].

The most prevalent category of chemicals detected with rapid comprehensive chemical analysis was sterols, most of which have a plant origin (phystosterol). However, other sterols such as coprostanol, epicoprostanol, cholesterol, 3-cholestanone, and cholestanol are of faecal origin [36] and found in mammalians [37,38,39]. Phytosterol oxide concentrates containing approximately 30% phytosterol oxides did not demonstrate genotoxic potential or evidence of toxicity and can be considered low risk [40]. The other prevalent group detected by rapid comprehensive chemical analysis was alkanes (‘paraffins’). The alkanes detected (C15 to C33) can either be produced by plants [41] or from oil refining to make waxes, anticorrosive agents, and lubricating oils [42].

The data confirm that the Napier sewage treatment plant had minimum input as a source of contaminants. This is consistent with microbial monitoring conducted that reported the highest concentrations of faecal indicator bacteria were detected in the proximity of the sewage treatment site [43].

## 5. Conclusions

Overall, the results from the toxicity bioassays did not suggest high risk in relation to the displaced sediment in the Waitangi catchment. We tested elutriates, which are complex mixtures of chemicals that are water-soluble and indicate bioavailability. The Microtox^®^ bioassay results showed some bacterial growth stimulation, which suggests that some components in the elutriate samples were beneficial nutrients. The most toxic sediment elutriate was found at Site 4679 with the blue mussel test, but the variation in pH could have been an influencing factor on the survival of the embryos. The yeast assays showed activities similar to what has been reported in Japan after the event of Typhoon Hagibis [11]. The relatively low toxicity of sediment following a high flood event has been reported previously. Ecotoxicity testing of soil samples in an area impacted by flooding caused by Hurricane Katrina reported no acute toxicity in soil or porewater [44]. It also confirmed that the level and type of contaminants displaced is correlated with the proximity of contaminated sites [8].

Rapid comprehensive chemical analysis is a qualitative method used to provide information on the presence of molecules, while the targeted analysis allows the quantification but only for the specific chemicals for which a reference is used. Although the rapid analysis’ sensitivity was lower than that of the targeted analysis, the quantitative values of DDT and pesticides detected by both methods were in agreement, indicating the excellent quantitative performance of AIQS. Both methods highlighted the presence of historical contamination by DDT and its metabolites DDE and DDD. These chemicals can, in part, contribute to the effects in the AhR bioassay. The concentrations of metals and metalloids tested were all below the Australia and New Zealand DGVs. There was also indication of mammalian faecal contamination, likely from farm animals. Overall, the results from this study suggest that the risk of the displaced sediment in the Waitangi catchment is low. However, some caution is warranted for handling and disposing of the sediment material.

## Figures and Tables

**Table 1:** Conditions of scoring in AIQS-GC. The number of asterisks (*) provides an indication of the accuracy of chemical identification. RI = retention index; MS hit rate = similarity of MS spectrum between AIQS-DB and samples; QT ratio = qualifier ion/target ion. These are calculated from the differences between actual results in the sample and predicted results in AIQS-DB. When the targets are evaluated for scoring, the ignored index is not considered.

Score	RI	MS Hit Rate (%)	QT Ratio
*****	−10 to +10	>25	Not considered
****	−20 to +20	>25	Not considered
***	−10 to +10	<25	0.9 to 1.1
**	−20 to +20	<25	0.8 to 1.2
*	−20 to +20	<25	<0.8 or >1.2

**Table 2:** AhR- and CAR-mediated activities measured in the sediment extracts using the Y2 H reporter gene assays using β-naphthoflavone (βna) and 4-tertoctyl-phenol (4 op) as reference toxicants.

Site	AhR (µg-βna eq./kg-dw)	CAR (µg-4 op eq./kg-dw)
4679	427	140
4680	349	34
4681	502	145

**Table 3:** Ecotoxicity parameters for the blue mussel 48 h embryo–larval development assay after exposure to the elutriates of the three sediment samples.

Site	LC_10_ (%) (95%CI)	LC_25_ (%) (95%CI)	LC_50_ (%) (95%CI)	N(S)EC (%) (95%CI)	NOEC	LOEC
4679	5.5 (4.4–6.6)	7.5 (6.5–8.4)	9.7 (9.0–10.4)	10.7 (7.9–12.2)	6.25	12.5
4680	9.6 (8.6–10.7)	11.9 (11.3–12.6)	14.4 (13.7–15.2)	12.3 (11.4–22.4)	6.25	12.5
4681	42.6 (38.3–47.0)	53.0 (49.1–56.9)	64.1 (60.5–67.7)	58.9 (46.2–95.7)	50	100

**Table 4:** Concentrations of total recoverable metals and metalloids (mg/kg dry weight) in the three sediment samples and their related default guideline values (DGVs).

Metal/Metalloid	Site 4679	Site 4680	Site 4681	DGV	DGV High
Arsenic	6.3	6.8	8.8	20	70
Cadmium	0.127	0.187	1.09	1.5	10
Chromium	21	20	22	80	370
Copper	10.4	8.7	15.9	65	270
Lead	13	13.3	12.4	50	220
Mercury	0.07	0.07	0.1	0.15	1
Nickel	14.7	15.4	14.2	21	52
Zinc	149	70	128	200	410

**Table 5:** Measured (Meas.) and normalised (Norm.) (at 1% TOC; Norm.) concentrations of organic contaminants (μg/kg dry weight) detected in the three sediment samples and their related default guideline values (DGVs). Numbers in bold indicate concentration values above the DGV—comparisons with DGVs are made with the normalised concentration value at 1% TOC.

	Site 4679		Site 4680		Site 4681		DGV	DGV High
	Meas.	Norm.	Meas.	Norm.	Meas.	Norm.		
Bifenthrin	33	**18.1**	<6		6	**2.5**	0.11	
2,4′-DDD	<1		22	15.2	28	11.7		
4,4′-DDD	4.4	2.4	12.9	8.9	111	46.3		
2,4′- + 4,4′-DDD	4.4	2.4	34.9	**24.1**	139	**57.9**	3.5	9
4,4′-DDE	4.2	**2.3**	25	**17.2**	20	**8.3**	1.4	7
2,4’-DDT	<1	–	1.6	1.1	<1	–		
4,4’-DDT	2.7	1.5	14.8	10.2	6.9	2.9		
Total DDT	2.7	**1.5**	16.4	**11.3**	6.9	**2.9**	1.2	5
Total DDT Isomers	12	6.6	56	38.6	230	95.8		
Dieldrin	<1	–	1.3	0.9	<1	–	2.8	7

## Data Availability

All data is available in the Supplementary Materials.

## Supplementary Materials

The following supporting information can be downloaded at: https://www.mdpi.com/article/10.3390/toxics12080558/s1, Figure S1: AIQS-GC screen, AXEL for NAGINATA spectrum for 4-nonylphenol.; Figure S2: Dose-response of *Aliivibrio fischeri* to an increasing concentration of the reference toxicant; Table S1: Composition of medium for pre-incubation and CAR final assay; Table S2: Components of the reaction solution; Table S3: Composition of medium for AhR final assay; Table S4: Summary of test conditions for the Microtox^®^ and blue mussel embryo-larval bioassays; Table S5: AIQS-GC measurement parameters; Table S6: Summary of data from the commercial analytical laboratory including targeted analyses of the soil samples; Table S7: Microtox^®^ raw data. Luminescence (l) and gamma (G) measured in the range of concentrations of the three sediment elutriates after 5, 15 and 30 min of exposure; Table S8: Physico-chemical parameters of the control and the three sediment elutriate samples at the start of the exposure at the highest tested concentrations for the blue mussel embryo-larval development assay; Table S9: Blue mussel larvae survival in sediment elutriate samples at a range of concentrations after 48 h of exposure; Table S10: Concentrations of the detected organic compounds by rapid comprehensive analysis in the three sediment samples collected.
