# Supplementary material for: Risk Assessment of Displaced Sediment by an Extreme Event Cyclone in a Peri-Urban Zone Using Bioassays and Analytical Chemistry
Source: Toxics. 2024 Jul 31;12(8):558. doi: 10.3390/toxics12080558 (PMC11360154; doi:10.3390/toxics12080558)

Supplementary Material- Toxics

Risk assessment of displaced sediment by an extreme event cyclone in a peri-urban zone using bioassays and analytical chemistry

Louis A. Tremblay [1,2,3*], Daisuke Nakajima [4] Satoshi Endo [4], Mayuko Yagishita [5], Hannah Ludlow [6,7], Ariana Mackay [6], Olivier Champeau [1]

[1] Cawthron Institute, Private Bag 2, Nelson 7042, New Zealand
[2] School of Biological Sciences, University of Auckland, Auckland 1142, New Zealand
[3] Manaaki Whenua- Landcare Research, Lincoln 7608, New Zealand
[4] Health and Environmental Risk Division, National Institute for Environmental Studies, Tsukuba, Japan
[5] Department of Life and Environmental Science, Prefectural University of Hiroshima, Hiroshima, Japan
[6] Hawke's Bay Regional Council, 159 Dalton Street, Napier 4110, New Zealand
[7] Pattle Delamore Partners, Ground Floor South, Bower House, 18 Bower Street, Napier, New Zealand
[*] Correspondence: tremblayl@landcareresearch.co.nz

Table S1. Composition of medium for pre-incubation and CAR final assay.

Yeast nitrogen base w/o amino acids		5.8 g
Dextrose		8.8 g
Dropout solution		174 mL
L-isoleucine	300 mg	
L-valine	1,500 mg	
L-adenine hemisulfate salt	200 mg	
L-arginine HCl	200 mg	
L-histidine HCl monohydrate	200 mg	
L-lysine HCl	300 mg	
L-methionine	200 mg	
L-phenylalanine	500 mg	
L-threonine	2,000 mg	
L-tyrosine	300 mg	
L-uracil	200 mg	
**Purified water**		**final 1,000 mL**

Table S2. Components of the reaction solution.

Solution A<br>200 μL	Galacton-Star（100× concentrated）	
Solution B<br>10 mL	100 mM sodium phosphate buffer (pH 7.4)	100 mL
	MgCl · $6H_2O$	0.02 g
	Sapphire-II	174 mL

Table S3. Composition of medium for AhR final assay.

Yeast nitrogen base w/o amino acids	5.8 g
Galactose	15 g
Dropout solution (-Trp)	174 mL
Purified water	final 1,000 mL

Table S4. Summary of test conditions for the Microtox® and blue mussel embryo-larval bioassays.

	**Microtox®**	**Mussel test**
Test start to end dates	7 June2023	14–16 June 2023
Standard	ISO 11348-3 (2007)	ASTM E724-21 (2021) ETX 4
Test species	*Aliivibrio fischeri*	*Mytilus galloprovincialis*
Source	BioLight Aqua-Science (Lot 10641121)	Pelorus Sound / Te Hoiere
Density, number per test container	n/a	~400
Type of test container	5 mL glass tube	6-well plate (10 mL/well)
Exposure time (hours)	0.08, 0.25, 0.5	48
Concentrations tested (%)	0, 0.2–50	0, 0.2–100
Replicates	2	5 for controls, 5 for treatments
Temperature (°C)	15	16.5 ±1.2
Dissolved oxygen (at the beginning of the test) (mg/L)	n/m	8.8 (109%)
pH	n/m	8.1
Dilution water	Type I with NaCl 2%	Reconstituted seawater
Aeration	None	None
Salinity (at the beginning of the test, PSU)	20	34.2
Endpoint	Luminescence	Survival
Sensitivity (EC_{50} with 95% confidence interval)	1.15(0.96–1.12) mg Zn^{2+}/L (15 minutes)	0.148 (0.144–0.153) mg Zn^{2+}/L
Control quality for sensitivity (mean ±2× standard deviation)	1.27 (0.98–1.56) mg Zn^{2+}/L (*n* = 3) (15 minutes)	0.174 (0.115–0.233) mg Zn^{2+}/L (*n* = 8)
Test acceptability (in controls)	Yes	No < 60% D-yield
Note	–	Collection date: 13 June 2023 Spawning method: thermal stimulation

Table S5. AIQS-GC measurement parameters

	Conditions
GC-MS	Capillary GC-Quadrupole MS
Column	Agilent (J&W) DB-5ms (30m, 0.25mm ID, 0.25μm FT)
Temperature	Oven: 40℃ (2min) →8℃/min → 310℃ (5 min)
	Injector: 250℃
	Transfer line: 280℃
	Ion source: 230℃
Injection method	Split less, purge off time: 1 min
Carrier gas	He
Carrier speed	1.2 ml/min
Ionization	EI
Tuning method	DFTPP target tuning (US EPA method 625)
Scan range	33 amu － 600amu
Scan speed	0.3s/scan

Table S6. Summary of data from the commercial analytical laboratory including targeted analyses of the soil samples.

R J Hill Laboratories Limited
28 Duke Street Frankton 3204
Private Bag 3205
Hamilton 3240 New Zealand
0508 HILL LAB (44 555 22)
+64 7 858 2000
mail@hill-labs.co.nz
www.hill-labs.co.nz

Certificate of Analysis

**Client:**	Hawkes Bay Regional Council	**Lab No:**	3280937 SPv3
**Contact:**	Hannah Ludlow	**Date Received:**	19-May-2023
	C/- Hawkes Bay Regional Council	**Date Reported:**	17-Jul-2023
	Private Bag 6006	**Quote No:**	122691
	Napier 4142	**Order No:**	PN00018761
		**Client Reference:**	Waitangi Sediment
		**Submitted By:**	Hannah Ludlow

Sample Type: Sediment

Sample Name:		102127 - Waitangi Stream opposite 32 Waitangi Rd: 4679 18-May-2023 1:30 pm	102129 - Waitangi Stream D/S BioRich driveway: 4680 18-May-2023 2:30 pm	102128 - Waitangi Stream D/S stopbank receiving environment: 4681 18-May-2023 3:00 pm
**Lab Number:**		3280937.1	3280937.2	3280937.3
Individual Tests				
Dry Matter	g/100g as rcvd	49	59	56
Total Recoverable Phosphorus	mg/kg dry wt	780	970	3,500
Total Recoverable Rubidium	mg/kg dry wt	17.8	17.7	14.8
Total Recoverable Silver	mg/kg dry wt	0.04	0.04	0.04
Total Cyanide*	mg/kg dry wt	< 0.10	< 0.10	< 0.10
Total Nitrogen*	g/100g dry wt	0.16	0.11	0.13
Total Organic Carbon*	g/100g dry wt	1.82	1.45	2.4
Heavy metals, trace As,Cd,Cr,Cu,Ni,Pb,Zn,Hg				
Total Recoverable Arsenic	mg/kg dry wt	6.3	6.8	8.8
Total Recoverable Cadmium	mg/kg dry wt	0.127	0.187	1.09
Total Recoverable Chromium	mg/kg dry wt	21	20	22
Total Recoverable Copper	mg/kg dry wt	10.4	8.7	15.9
Total Recoverable Lead	mg/kg dry wt	13.0	13.3	12.4
Total Recoverable Mercury	mg/kg dry wt	0.07	0.07	0.10
Total Recoverable Nickel	mg/kg dry wt	14.7	15.4	14.2
Total Recoverable Zinc	mg/kg dry wt	149	70	128
7 Grain Sizes Profile as received*				
Dry Matter of Sieved Sample*	g/100g as rcvd	50	60	48
Fraction >/= 2 mm*	g/100g dry wt	0.2	7.1	0.8
Fraction < 2 mm, >/= 1 mm*	g/100g dry wt	0.2	0.6	1.0
Fraction < 1 mm, >/= 500 µm*	g/100g dry wt	0.1	0.9	1.2
Fraction < 500 µm, >/= 250 µm*	g/100g dry wt	0.2	1.1	2.7
Fraction < 250 µm, >/= 125 µm*	g/100g dry wt	0.4	3.6	11.6
Fraction < 125 µm, >/= 63 µm*	g/100g dry wt	2.2	17.1	29.1
Fraction < 63 µm*	g/100g dry wt	96.7	69.7	53.6
Acid Herbicides Trace in Soil by LCMSMS*				
Acifluorfen	mg/kg dry wt	< 0.010	< 0.010	< 0.010
Bentazone	mg/kg dry wt	< 0.010	< 0.010	< 0.010
Bromoxynil	mg/kg dry wt	< 0.010	< 0.010	< 0.010
Clopyralid	mg/kg dry wt	< 0.010	< 0.010	< 0.010
Dicamba	mg/kg dry wt	< 0.010	< 0.010	< 0.010
2,4-Dichlorophenoxyacetic acid (24D)	mg/kg dry wt	< 0.010	< 0.010	< 0.010
2,4-Dichlorophenoxybutyric acid (24DB)	mg/kg dry wt	< 0.010	< 0.010	< 0.010
Dichlorprop	mg/kg dry wt	< 0.010	< 0.010	< 0.010

This Laboratory is accredited by International Accreditation New Zealand (IANZ), which represents New Zealand in the International Laboratory Accreditation Cooperation (ILAC). Through the ILAC Mutual Recognition Arrangement (ILAC-MRA) this accreditation is internationally recognised.
The tests reported herein have been performed in accordance with the terms of accreditation, with the exception of tests marked * or any comments and interpretations, which are not accredited.

Table S6- continued.

Sample Type: Sediment				
**Sample Name:**		102127 - Waitangi Stream opposite 32 Waitangi Rd: 4679 18-May-2023 1:30 pm	102129 - Waitangi Stream D/S BioRich driveway: 4680 18-May-2023 2:30 pm	102128 - Waitangi Stream D/S stopbank receiving environment: 4681 18-May-2023 3:00 pm
**Lab Number:**		3280937.1	3280937.2	3280937.3
Acid Herbicides Trace in Soil by LCMSMS*				
Fluazifop	mg/kg dry wt	< 0.010	< 0.010	< 0.010
Fluroxypyr	mg/kg dry wt	< 0.010	< 0.010	< 0.010
Haloxyfop	mg/kg dry wt	< 0.010	< 0.010	< 0.010
2-methyl-4-chlorophenoxyacetic acid (MCPA)	mg/kg dry wt	< 0.010	< 0.010	< 0.010
2-methyl-4-chlorophenoxybutanoic acid (MCPB)	mg/kg dry wt	< 0.010	< 0.010	< 0.010
Mecoprop (MCPP; 2-methyl-4-chlorophenoxypropionic acid)	mg/kg dry wt	< 0.010	< 0.010	< 0.010
Oryzalin	mg/kg dry wt	< 0.02	< 0.02	< 0.02
Pentachlorophenol (PCP)	mg/kg dry wt	< 0.010	< 0.010	< 0.010
Picloram	mg/kg dry wt	< 0.010	< 0.010	< 0.010
Quizalofop	mg/kg dry wt	< 0.010	< 0.010	< 0.010
2,3,4,6-Tetrachlorophenol (TCP)	mg/kg dry wt	< 0.010	< 0.010	< 0.010
2,4,5-trichlorophenoxypropionic acid (245TP,Fenoprop, Silvex)	mg/kg dry wt	< 0.010	< 0.010	< 0.010
2,4,5-Trichlorophenoxyacetic acid (245T)	mg/kg dry wt	< 0.010	< 0.010	< 0.010
Triclopyr*	mg/kg dry wt	< 0.010	< 0.010	< 0.010
Multiresidue Pesticides in Sediment samples by GCMS				
Acetochlor	mg/kg dry wt	< 0.014	< 0.011	< 0.012
Alachlor	mg/kg dry wt	< 0.007	< 0.006	< 0.006
Atrazine	mg/kg dry wt	< 0.014	< 0.011	< 0.012
Atrazine-desethyl	mg/kg dry wt	< 0.014	< 0.011	< 0.012
Atrazine-desisopropyl	mg/kg dry wt	< 0.03	< 0.03	< 0.03
Azaconazole	mg/kg dry wt	< 0.007	< 0.006	< 0.006
Azinphos-methyl	mg/kg dry wt	< 0.03	< 0.03	< 0.03
Benalaxyl	mg/kg dry wt	< 0.007	< 0.006	< 0.006
Bendiocarb	mg/kg dry wt	< 0.014	< 0.011	< 0.012
Benodanil	mg/kg dry wt	< 0.03	< 0.03	< 0.03
Bifenthrin	mg/kg dry wt	0.033	< 0.006	0.006
Bitertanol	mg/kg dry wt	< 0.03	< 0.03	< 0.03
Bromacil	mg/kg dry wt	< 0.014	< 0.011	< 0.012
Bromophos-ethyl	mg/kg dry wt	< 0.014	< 0.011	< 0.012
Bromopropylate	mg/kg dry wt	< 0.014	< 0.011	< 0.012
Bupirimate	mg/kg dry wt	< 0.014	< 0.011	< 0.012
Buprofezin	mg/kg dry wt	< 0.014	< 0.011	< 0.012
Butachlor	mg/kg dry wt	< 0.014	< 0.011	< 0.12
Captafol	mg/kg dry wt	< 0.07	< 0.06	< 0.06
Captan	mg/kg dry wt	< 0.03	< 0.03	< 0.03
Carbaryl	mg/kg dry wt	< 0.014	< 0.011	< 0.012
Carbofenothion	mg/kg dry wt	< 0.014	< 0.011	< 0.012
Carbofuran	mg/kg dry wt	< 0.014	< 0.011	< 0.012
Chlorfenvinphos	mg/kg dry wt	< 0.019	< 0.016	< 0.017
Chlorfluazuron	mg/kg dry wt	< 0.014	< 0.011	< 0.012
Chlorothalonil	mg/kg dry wt	< 0.014	< 0.011	< 0.012
Chlorpropham	mg/kg dry wt	< 0.03	< 0.03	< 0.03
Chlorpyrifos	mg/kg dry wt	< 0.014	< 0.011	< 0.012
Chlorpyrifos-methyl	mg/kg dry wt	< 0.014	< 0.011	< 0.012
Chlortoluron	mg/kg dry wt	< 0.03	< 0.03	< 0.03
Chlozolinate	mg/kg dry wt	< 0.014	< 0.011	< 0.12
Coumaphos	mg/kg dry wt	< 0.03	< 0.03	< 0.03
Cyanazine	mg/kg dry wt	< 0.014	< 0.011	< 0.12
Cyfluthrin	mg/kg dry wt	< 0.016	< 0.014	< 0.015

Table S6- continued.

Sample Type: Sediment				
**Sample Name:**		102127 - Waitangi Stream opposite 32 Waitangi Rd: 4679 18-May-2023 1:30 pm	102129 - Waitangi Stream D/S BioRich driveway: 4680 18-May-2023 2:30 pm	102128 - Waitangi Stream D/S stopbank receiving environment: 4681 18-May-2023 3:00 pm
**Lab Number:**		3280937.1	3280937.2	3280937.3
Multiresidue Pesticides in Sediment samples by GCMS				
Cyhalothrin	mg/kg dry wt	< 0.014	< 0.011	< 0.012
Cypermethrin	mg/kg dry wt	< 0.04	< 0.03	< 0.03
Cyproconazole	mg/kg dry wt	< 0.03	< 0.03	< 0.03
Cyprodinil	mg/kg dry wt	< 0.014	< 0.011	< 0.12
Deltamethrin (including Tralomethrin)	mg/kg dry wt	< 0.014	< 0.011	< 0.012
Diazinon	mg/kg dry wt	< 0.007	< 0.006	< 0.006
Dichlobenil	mg/kg dry wt	< 0.014	< 0.011	< 0.012
Dichlofenthion	mg/kg dry wt	< 0.014	< 0.011	< 0.012
Dichlofluanid	mg/kg dry wt	< 0.014	< 0.011	< 0.012
Dichloran	mg/kg dry wt	< 0.04	< 0.03	< 0.03
Dichlorvos	mg/kg dry wt	< 0.014	< 0.011	< 0.012
Dicofol	mg/kg dry wt	< 0.07	< 0.06	< 0.06
Dicrotophos	mg/kg dry wt	< 0.014	< 0.011	< 0.012
Difenoconazole	mg/kg dry wt	< 0.019	< 0.016	< 0.017
Dimethoate	mg/kg dry wt	< 0.03	< 0.03	< 0.03
Dinocap	mg/kg dry wt	< 0.15	< 0.13	< 0.13
Diphenylamine	mg/kg dry wt	< 0.03	< 0.03	< 0.03
Diuron	mg/kg dry wt	< 0.014	< 0.011	< 0.012
EPN	mg/kg dry wt	< 0.014	< 0.011	< 0.012
Ethion	mg/kg dry wt	< 0.014	< 0.011	< 0.012
Etrimfos	mg/kg dry wt	< 0.014	< 0.011	< 0.012
Famphur	mg/kg dry wt	< 0.014	< 0.011	< 0.012
Fenarimol	mg/kg dry wt	< 0.014	< 0.011	< 0.012
Fenitrothion	mg/kg dry wt	< 0.014	< 0.011	< 0.012
Fenpropathrin	mg/kg dry wt	< 0.014	< 0.011	< 0.012
Fenpropimorph	mg/kg dry wt	< 0.014	< 0.011	< 0.12
Fensulfothion	mg/kg dry wt	< 0.014	< 0.011	< 0.012
Fenvalerate (including Esfenvalerate)	mg/kg dry wt	< 0.019	< 0.016	< 0.017
Fluazifop-butyl	mg/kg dry wt	< 0.014	< 0.011	< 0.012
Fluometuron	mg/kg dry wt	< 0.014	< 0.011	< 0.012
Flusilazole	mg/kg dry wt	< 0.014	< 0.011	< 0.012
Fluvalinate	mg/kg dry wt	< 0.010	< 0.008	< 0.009
Folpet	mg/kg dry wt	< 0.03	< 0.03	< 0.3
Furalaxyl	mg/kg dry wt	< 0.007	< 0.006	< 0.006
Haloxyfop-methyl	mg/kg dry wt	< 0.014	< 0.011	< 0.012
Hexaconazole	mg/kg dry wt	< 0.014	< 0.011	< 0.012
Hexazinone	mg/kg dry wt	< 0.007	< 0.006	< 0.006
Hexythiazox	mg/kg dry wt	< 0.07	< 0.06	< 0.06
Imazalil	mg/kg dry wt	< 0.07	< 0.06	< 0.06
Indoxacarb	mg/kg dry wt	< 0.014	< 0.011	< 0.012
Iodofenphos	mg/kg dry wt	< 0.014	< 0.011	< 0.012
IPBC (3-Iodo-2-propynyl-n-butylcarbamate)	mg/kg dry wt	< 0.07	< 0.06	< 0.06
Isazophos	mg/kg dry wt	< 0.014	< 0.011	< 0.012
Isofenphos	mg/kg dry wt	< 0.007	< 0.006	< 0.006
Kresoxim-methyl	mg/kg dry wt	< 0.007	< 0.006	< 0.006
Leptophos	mg/kg dry wt	< 0.014	< 0.011	< 0.012
Linuron	mg/kg dry wt	< 0.014	< 0.011	< 0.012
Malathion	mg/kg dry wt	< 0.014	< 0.011	< 0.012
Metalaxyl	mg/kg dry wt	< 0.014	< 0.011	< 0.012
Methacrifos	mg/kg dry wt	< 0.014	< 0.011	< 0.012
Methamidophos	mg/kg dry wt	< 0.07	< 0.06	< 0.06

Table S6- continued.

Sample Type: Sediment				
**Sample Name:**		102127 - Waitangi Stream opposite 32 Waitangi Rd: 4679 18-May-2023 1:30 pm	102129 - Waitangi Stream D/S BioRich driveway: 4680 18-May-2023 2:30 pm	102128 - Waitangi Stream D/S stopbank receiving environment: 4681 18-May-2023 3:00 pm
**Lab Number:**		3280937.1	3280937.2	3280937.3
Multiresidue Pesticides in Sediment samples by GCMS				
Methidathion	mg/kg dry wt	< 0.014	< 0.011	< 0.012
Methiocarb	mg/kg dry wt	< 0.014	< 0.011	< 0.012
Metolachlor	mg/kg dry wt	< 0.007	< 0.006	< 0.06
Metribuzin	mg/kg dry wt	< 0.014	< 0.011	< 0.012
Mevinphos	mg/kg dry wt	< 0.03	< 0.03	< 0.03
Molinate	mg/kg dry wt	< 0.03	< 0.03	< 0.03
Myclobutanil	mg/kg dry wt	< 0.014	< 0.011	< 0.012
Naled	mg/kg dry wt	< 0.07	< 0.06	< 0.06
Nitrofen	mg/kg dry wt	< 0.03	< 0.03	< 0.03
Nitrothal-isopropyl	mg/kg dry wt	< 0.014	< 0.011	< 0.012
Norflurazon	mg/kg dry wt	< 0.03	< 0.03	< 0.03
Omethoate	mg/kg dry wt	< 0.07	< 0.06	< 0.06
Oxadiazon	mg/kg dry wt	< 0.014	< 0.011	< 0.012
Oxychlordane	mg/kg dry wt	< 0.007	< 0.006	< 0.006
Oxyfluorfen	mg/kg dry wt	< 0.007	< 0.006	< 0.006
Paclobutrazol	mg/kg dry wt	< 0.014	< 0.011	< 0.012
Parathion-ethyl	mg/kg dry wt	< 0.014	< 0.011	< 0.012
Parathion-methyl	mg/kg dry wt	< 0.014	< 0.011	< 0.012
Penconazole	mg/kg dry wt	< 0.014	< 0.011	< 0.012
Pendimethalin	mg/kg dry wt	< 0.014	< 0.011	< 0.012
Permethrin	mg/kg dry wt	0.40	< 0.004	< 0.004
Phosmet	mg/kg dry wt	< 0.014	< 0.011	< 0.012
Phosphamidon	mg/kg dry wt	< 0.014	< 0.011	< 0.012
Pirimicarb	mg/kg dry wt	< 0.014	< 0.011	< 0.012
Pirimiphos-methyl	mg/kg dry wt	< 0.014	< 0.011	< 0.012
Prochloraz	mg/kg dry wt	< 0.07	< 0.06	< 0.06
Procymidone	mg/kg dry wt	< 0.014	< 0.011	< 0.12
Prometryn	mg/kg dry wt	< 0.007	< 0.006	< 0.006
Propachlor	mg/kg dry wt	< 0.014	< 0.011	< 0.012
Propanil	mg/kg dry wt	< 0.03	< 0.03	< 0.03
Propazine	mg/kg dry wt	< 0.007	< 0.006	< 0.006
Propetamphos	mg/kg dry wt	< 0.014	< 0.011	< 0.012
Propham	mg/kg dry wt	< 0.014	< 0.011	< 0.012
Propiconazole	mg/kg dry wt	< 0.010	< 0.008	< 0.009
Prothiofos	mg/kg dry wt	< 0.014	< 0.011	< 0.012
Pyrazophos	mg/kg dry wt	< 0.014	< 0.011	< 0.012
Pyrifenox	mg/kg dry wt	< 0.019	< 0.016	< 0.17
Pyrimethanil	mg/kg dry wt	< 0.014	< 0.011	< 0.012
Pyriproxyfen	mg/kg dry wt	< 0.014	< 0.011	< 0.012
Quintozene	mg/kg dry wt	< 0.03	< 0.03	< 0.03
Quizalofop-ethyl	mg/kg dry wt	< 0.014	< 0.011	< 0.012
Simazine	mg/kg dry wt	< 0.014	< 0.011	< 0.012
Simetryn	mg/kg dry wt	< 0.014	< 0.011	< 0.012
Sulfentrazone	mg/kg dry wt	< 0.07	< 0.06	< 0.06
Sulfotep	mg/kg dry wt	< 0.014	< 0.011	< 0.012
TCMTB [2-(thiocyanomethylthio) benzothiazole,Busan]	mg/kg dry wt	< 0.03	< 0.03	< 0.03
Tebuconazole	mg/kg dry wt	< 0.014	< 0.011	< 0.012
Tebufenpyrad	mg/kg dry wt	< 0.007	< 0.006	< 0.006
Terbacil	mg/kg dry wt	< 0.014	< 0.011	< 0.012
Terbumeton	mg/kg dry wt	< 0.014	< 0.011	< 0.012
Terbuthylazine	mg/kg dry wt	< 0.007	< 0.006	< 0.006
Terbuthylazine-desethyl	mg/kg dry wt	< 0.014	< 0.011	< 0.012
Terbutryn	mg/kg dry wt	< 0.014	< 0.011	< 0.12

Table S6- continued.

Sample Type: Sediment				
**Sample Name:**		102127 - Waitangi Stream opposite 32 Waitangi Rd: 4679 18-May-2023 1:30 pm	102129 - Waitangi Stream D/S BioRich driveway: 4680 18-May-2023 2:30 pm	102128 - Waitangi Stream D/S stopbank receiving environment: 4681 18-May-2023 3:00 pm
**Lab Number:**		3280937.1	3280937.2	3280937.3
Multiresidue Pesticides in Sediment samples by GCMS				
Tetrachlorvinphos	mg/kg dry wt	< 0.014	< 0.011	< 0.012
Thiabendazole	mg/kg dry wt	< 0.07	< 0.06	< 0.06
Thiobencarb	mg/kg dry wt	< 0.014	< 0.011	< 0.12
Tolylfluanid	mg/kg dry wt	< 0.007	< 0.006	< 0.006
Triadimefon	mg/kg dry wt	< 0.014	< 0.011	< 0.012
Triazophos	mg/kg dry wt	< 0.014	< 0.011	< 0.012
Trifluralin	mg/kg dry wt	< 0.014	< 0.011	< 0.012
Vinclozolin	mg/kg dry wt	< 0.014	< 0.011	< 0.012
Organochlorine Pesticides Trace in Soil				
Aldrin	mg/kg dry wt	< 0.0010	< 0.0010	< 0.0010
alpha-BHC	mg/kg dry wt	< 0.0010	< 0.0010	< 0.0010
beta-BHC	mg/kg dry wt	< 0.0010	< 0.0010	< 0.0010
delta-BHC	mg/kg dry wt	< 0.0010	< 0.0010	< 0.0010
gamma-BHC (Lindane)	mg/kg dry wt	< 0.0010	< 0.0010	< 0.0010
cis-Chlordane	mg/kg dry wt	< 0.0010	< 0.0010	< 0.0010
trans-Chlordane	mg/kg dry wt	< 0.0010	< 0.0010	< 0.0010
2,4'-DDD	mg/kg dry wt	< 0.0010	0.0022	0.028
4,4'-DDD	mg/kg dry wt	0.0044	0.0129	0.111
2,4'-DDE	mg/kg dry wt	< 0.0010	< 0.0010	< 0.0010
4,4'-DDE	mg/kg dry wt	0.0042	0.025	0.020
2,4'-DDT	mg/kg dry wt	< 0.0010	0.0016	< 0.0010
4,4'-DDT	mg/kg dry wt	0.0027	0.0148	0.069
Total DDT Isomers	mg/kg dry wt	0.012	0.056	0.23
Dieldrin	mg/kg dry wt	< 0.0010	0.0013	< 0.0010
Endosulfan I	mg/kg dry wt	< 0.0010	< 0.0010	< 0.0010
Endosulfan II	mg/kg dry wt	< 0.0010	< 0.0010	< 0.0010
Endosulfan sulphate	mg/kg dry wt	< 0.0010	< 0.0010	< 0.0010
Endrin	mg/kg dry wt	< 0.0010	< 0.0010	< 0.0010
Endrin aldehyde	mg/kg dry wt	< 0.0010	< 0.0010	< 0.0010
Endrin ketone	mg/kg dry wt	< 0.0010	< 0.0010	< 0.0010
Heptachlor	mg/kg dry wt	< 0.0010	< 0.0010	< 0.0010
Heptachlor epoxide	mg/kg dry wt	< 0.0010	< 0.0010	< 0.0010
Hexachlorobenzene	mg/kg dry wt	< 0.0010	< 0.0010	< 0.0010
Methoxychlor	mg/kg dry wt	< 0.0010	< 0.0010	< 0.0010
Polychlorinated Biphenyls Trace in Soil*				
PCB-18	mg/kg dry wt	< 0.0010	< 0.0010	< 0.0010
PCB-28	mg/kg dry wt	< 0.0010	< 0.0010	< 0.0010
PCB-31	mg/kg dry wt	< 0.0010	< 0.0010	< 0.0010
PCB-44	mg/kg dry wt	< 0.0010	< 0.0010	< 0.0010
PCB-49	mg/kg dry wt	< 0.0010	< 0.0010	< 0.0010
PCB-52	mg/kg dry wt	< 0.0010	< 0.0010	< 0.0010
PCB-60	mg/kg dry wt	< 0.0010	< 0.0010	< 0.0010
PCB-77	mg/kg dry wt	< 0.0010	< 0.0010	< 0.0010
PCB-81	mg/kg dry wt	< 0.0010	< 0.0010	< 0.0010
PCB-86	mg/kg dry wt	< 0.0010	< 0.0010	< 0.0010
PCB-101	mg/kg dry wt	< 0.0010	< 0.0010	< 0.0010
PCB-105	mg/kg dry wt	< 0.0010	< 0.0010	< 0.0010
PCB-110	mg/kg dry wt	< 0.0010	< 0.0010	< 0.0010
PCB-114	mg/kg dry wt	< 0.0010	< 0.0010	< 0.0010
PCB-118	mg/kg dry wt	< 0.0010	< 0.0010	< 0.0010
PCB-121	mg/kg dry wt	< 0.0010	< 0.0010	< 0.0010
PCB-123	mg/kg dry wt	< 0.0010	< 0.0010	< 0.0010
PCB-126	mg/kg dry wt	< 0.0010	< 0.0010	< 0.0010

Table S6- continued.

Sample Type: Sediment				
Sample Name:		102127 - Waitangi Stream opposite 32 Waitangi Rd: 4679 18-May-2023 1:30 pm	102129 - Waitangi Stream D/S BioRich driveway: 4680 18-May-2023 2:30 pm	102128 - Waitangi Stream D/S stopbank receiving environment: 4681 18-May-2023 3:00 pm
Lab Number:		3280937.1	3280937.2	3280937.3
Polychlorinated Biphenyls Trace in Soil*				
PCB-128	mg/kg dry wt	< 0.0010	< 0.0010	< 0.0010
PCB-138	mg/kg dry wt	< 0.0010	< 0.0010	< 0.0010
PCB-141	mg/kg dry wt	< 0.0010	< 0.0010	< 0.0010
PCB-149	mg/kg dry wt	< 0.0010	< 0.0010	< 0.0010
PCB-151	mg/kg dry wt	< 0.0010	< 0.0010	< 0.0010
PCB-153	mg/kg dry wt	< 0.0010	< 0.0010	< 0.0010
PCB-156	mg/kg dry wt	< 0.0010	< 0.0010	< 0.0010
PCB-157	mg/kg dry wt	< 0.0010	< 0.0010	< 0.0010
PCB-159	mg/kg dry wt	< 0.0010	< 0.0010	< 0.0010
PCB-167	mg/kg dry wt	< 0.0010	< 0.0010	< 0.0010
PCB-169	mg/kg dry wt	< 0.0010	< 0.0010	< 0.0010
PCB-170	mg/kg dry wt	< 0.0010	< 0.0010	< 0.0010
PCB-180	mg/kg dry wt	< 0.0010	< 0.0010	< 0.0010
PCB-189	mg/kg dry wt	< 0.0010	< 0.0010	< 0.0010
PCB-194	mg/kg dry wt	< 0.0010	< 0.0010	< 0.0010
PCB-206	mg/kg dry wt	< 0.0010	< 0.0010	< 0.0010
PCB-209	mg/kg dry wt	< 0.0010	< 0.0010	< 0.0010
Mono-Ortho PCB Toxic Equivalence (TEF)*	mg/kg dry wt	< 0.0000003	< 0.0000003	< 0.0000003
Non-Ortho PCB Toxic Equivalence (TEF)*	mg/kg dry wt	< 0.0002	< 0.0002	< 0.0002
Total PCB (Sum of 35 congeners)	mg/kg dry wt	< 0.035	< 0.035	< 0.035
Haloethers Trace in SVOC Soil Samples by GC-MS				
Bis(2-chloroethoxy) methane	mg/kg dry wt	< 0.12	< 0.10	< 0.11
Bis(2-chloroethyl)ether	mg/kg dry wt	< 0.12	< 0.10	< 0.11
Bis(2-chloroisopropyl)ether	mg/kg dry wt	< 0.12	< 0.10	< 0.11
4-Bromophenyl phenyl ether	mg/kg dry wt	< 0.12	< 0.10	< 0.11
4-Chlorophenyl phenyl ether	mg/kg dry wt	< 0.12	< 0.10	< 0.11
Nitrogen containing compounds Trace in SVOC Soil Samples, GC-MS				
N-Nitrosodiphenylamine + Diphenylamine	mg/kg dry wt	< 0.3	< 0.2	< 0.3
2,4-Dinitrotoluene	mg/kg dry wt	< 0.3	< 0.2	< 0.3
2,6-Dinitrotoluene	mg/kg dry wt	< 0.3	< 0.2	< 0.3
Nitrobenzene	mg/kg dry wt	< 0.12	< 0.10	< 0.11
N-Nitrosodi-n-propylamine	mg/kg dry wt	< 0.3	< 0.2	< 0.3
Organochlorine Pesticides Trace in SVOC Soil Samples by GC-MS				
Aldrin	mg/kg dry wt	< 0.12	< 0.10	< 0.11
alpha-BHC	mg/kg dry wt	< 0.12	< 0.10	< 0.11
beta-BHC	mg/kg dry wt	< 0.12	< 0.10	< 0.11
delta-BHC	mg/kg dry wt	< 0.12	< 0.10	< 0.11
gamma-BHC (Lindane)	mg/kg dry wt	< 0.12	< 0.10	< 0.11
4,4'-DDD	mg/kg dry wt	< 0.12	< 0.10	< 0.11
4,4'-DDE	mg/kg dry wt	< 0.12	< 0.10	< 0.11
4,4'-DDT	mg/kg dry wt	< 0.3	< 0.2	< 0.3
Dieldrin	mg/kg dry wt	< 0.12	< 0.10	< 0.11
Endosulfan I	mg/kg dry wt	< 0.3	< 0.2	< 0.3
Endosulfan II	mg/kg dry wt	< 0.5	< 0.5	< 0.5
Endosulfan sulphate	mg/kg dry wt	< 0.3	< 0.2	< 0.3
Endrin	mg/kg dry wt	< 0.3	< 0.2	< 0.3
Endrin ketone	mg/kg dry wt	< 0.3	< 0.2	< 0.3
Heptachlor	mg/kg dry wt	< 0.12	< 0.10	< 0.11
Heptachlor epoxide	mg/kg dry wt	< 0.12	< 0.10	< 0.11
Hexachlorobenzene	mg/kg dry wt	< 0.12	< 0.10	< 0.11

Table S6- continued.

Sample Type: Sediment				
**Sample Name:**		102127 - Waitangi Stream opposite 32 Waitangi Rd: 4679 18-May-2023 1:30 pm	102129 - Waitangi Stream D/S BioRich driveway: 4680 18-May-2023 2:30 pm	102128 - Waitangi Stream D/S stopbank receiving environment: 4681 18-May-2023 3:00 pm
**Lab Number:**		3280937.1	3280937.2	3280937.3
Polycyclic Aromatic Hydrocarbons Trace in SVOC Soil Samples*				
Acenaphthene	mg/kg dry wt	< 0.10	< 0.10	< 0.10
Acenaphthylene	mg/kg dry wt	< 0.10	< 0.10	< 0.10
Anthracene	mg/kg dry wt	< 0.10	< 0.10	< 0.10
Benzo[a]anthracene	mg/kg dry wt	< 0.10	< 0.10	< 0.10
Benzo[a]pyrene (BAP)	mg/kg dry wt	< 0.12	< 0.10	< 0.11
Benzo[b]fluoranthene + Benzo[j] fluoranthene	mg/kg dry wt	< 0.12	< 0.10	< 0.11
Benzo[g,h,i]perylene	mg/kg dry wt	< 0.12	< 0.10	< 0.11
Benzo[k]fluoranthene	mg/kg dry wt	< 0.12	< 0.10	< 0.11
1&2-Chloronaphthalene	mg/kg dry wt	< 0.10	< 0.10	< 0.10
Chrysene	mg/kg dry wt	< 0.10	< 0.10	< 0.10
Dibenzo[a,h]anthracene	mg/kg dry wt	< 0.12	< 0.10	< 0.11
Fluoranthene	mg/kg dry wt	< 0.10	< 0.10	< 0.10
Fluorene	mg/kg dry wt	< 0.10	< 0.10	< 0.10
Indeno(1,2,3-c,d)pyrene	mg/kg dry wt	< 0.12	< 0.10	< 0.11
2-Methylnaphthalene	mg/kg dry wt	< 0.10	< 0.10	< 0.10
Naphthalene	mg/kg dry wt	< 0.10	< 0.10	< 0.10
Phenanthrene	mg/kg dry wt	< 0.10	< 0.10	< 0.10
Pyrene	mg/kg dry wt	< 0.10	< 0.10	< 0.10
Benzo[a]pyrene Potency Equivalency Factor (PEF) NES*	mg/kg dry wt	< 0.29	< 0.25	< 0.26
Benzo[a]pyrene Toxic Equivalence (TEF)*	mg/kg dry wt	< 0.29	< 0.25	< 0.25
Phenols Trace in SVOC Soil Samples by GC-MS				
4-Chloro-3-methylphenol	mg/kg dry wt	< 0.5	< 0.5	< 0.5
2-Chlorophenol	mg/kg dry wt	< 0.2	< 0.2	< 0.2
2,4-Dichlorophenol	mg/kg dry wt	< 0.2	< 0.2	< 0.2
2,4-Dimethylphenol	mg/kg dry wt	< 0.4	< 0.4	< 0.4
3 & 4-Methylphenol (m- + p-cresol)	mg/kg dry wt	< 0.4	< 0.4	< 0.4
2-Methylphenol (o-cresol)	mg/kg dry wt	< 0.2	< 0.2	< 0.2
2-Nitrophenol	mg/kg dry wt	< 0.4	< 0.4	< 0.4
Pentachlorophenol (PCP)	mg/kg dry wt	< 6	< 6	< 6
Phenol	mg/kg dry wt	< 0.3	< 0.2	< 0.3
2,4,5-Trichlorophenol	mg/kg dry wt	< 0.3	< 0.2	< 0.3
2,4,6-Trichlorophenol	mg/kg dry wt	< 0.3	< 0.2	< 0.3
Plasticisers Trace in SVOC Soil Samples by GC-MS				
Bis(2-ethylhexyl)phthalate	mg/kg dry wt	1.3	< 0.5	< 0.5
Butylbenzylphthalate	mg/kg dry wt	< 0.3	< 0.2	< 0.3
Di(2-ethylhexyl)adipate	mg/kg dry wt	< 0.2	< 0.2	< 0.2
Diethylphthalate	mg/kg dry wt	< 0.3	< 0.2	< 0.3
Dimethylphthalate	mg/kg dry wt	< 0.3	< 0.2	< 0.3
Di-n-butylphthalate	mg/kg dry wt	< 0.3	< 0.2	< 0.3
Di-n-octylphthalate	mg/kg dry wt	< 0.3	< 0.2	< 0.3
Other Halogenated compounds Trace in SVOC Soil Samples by GC-MS				
1,2-Dichlorobenzene	mg/kg dry wt	< 0.3	< 0.2	< 0.3
1,3-Dichlorobenzene	mg/kg dry wt	< 0.3	< 0.2	< 0.3
1,4-Dichlorobenzene	mg/kg dry wt	< 0.3	< 0.2	< 0.3
Hexachlorobutadiene	mg/kg dry wt	< 0.3	< 0.2	< 0.3
Hexachloroethane	mg/kg dry wt	< 0.3	< 0.2	< 0.3
1,2,4-Trichlorobenzene	mg/kg dry wt	< 0.12	< 0.10	< 0.11
Other SVOC Trace in SVOC Soil Samples by GC-MS				
Benzyl alcohol	mg/kg dry wt	< 1.2	< 1.0	< 1.1
Carbazole	mg/kg dry wt	< 0.12	< 0.10	< 0.11

Table S6- continued.

**Sample Type: Sediment**				
**Sample Name:**		102127 - Waitangi Stream opposite 32 Waitangi Rd: 4679 18-May-2023 1:30 pm	102129 - Waitangi Stream D/S BioRich driveway: 4680 18-May-2023 2:30 pm	102128 - Waitangi Stream D/S stopbank receiving environment: 4681 18-May-2023 3:00 pm
**Lab Number:**		3280937.1	3280937.2	3280937.3
Other SVOC Trace in SVOC Soil Samples by GC-MS				
Dibenzofuran	mg/kg dry wt	< 0.12	< 0.10	< 0.11
Isophorone	mg/kg dry wt	< 0.12	< 0.10	< 0.11
Total Petroleum Hydrocarbons in Solids				
C7 - C9	mg/kg dry wt	< 30	< 30	< 30
C10 - C14	mg/kg dry wt	< 30	< 20	< 30
C15 - C36	mg/kg dry wt	74	< 40	< 50
Total hydrocarbons (C7 - C36)	mg/kg dry wt	< 100	< 90	< 90

3280937.1
102127 - Waitangi Stream opposite 32 Waitangi Rd: 4679 18-May-2023 1:30 pm
Client Chromatogram for TPH by FID

Analyst's Comments

It has been noted that the System Monitoring Compounds 2-fluorophenol and phenol-d5 in the SVOC analysis on sample 3280937.3 had lower than expected recoveries. The recoveries were 38% and 38% respectively. Therefore the phenolic compounds may be underestimated.

The following table(s) gives a brief description of the methods used to conduct the analyses for this job. The detection limits given below are those attainable in a relatively simple matrix. Detection limits may be higher for individual samples should insufficient sample be available, or if the matrix requires that dilutions be performed during analysis. A detection limit range indicates the lowest and highest detection limits in the associated suite of analytes. A full listing of compounds and detection limits are available from the laboratory upon request. Unless otherwise indicated, analyses were performed at Hill Labs, 28 Duke Street, Frankton, Hamilton 3204.

Sample Type: Sediment

Test	Method Description	Default Detection Limit	Sample No
Individual Tests			
Environmental Solids Sample Drying*	Air dried at 35°C<br>Used for sample preparation.<br>May contain a residual moisture content of 2-5%.	-	1-3
Environmental Solids Sample Preparation	Air dried at 35°C and sieved, <2mm fraction.<br>Used for sample preparation<br>May contain a residual moisture content of 2-5%.	-	1-3
Soil Prep Dry for Organics,Trace*	Air dried at 35°C<br>Used for sample preparation.<br>May contain a residual moisture content of 2-5%.	-	1-3
Dry Matter	Dried at 103°C for 4-22hr (removes 3-5% more water than air dry) , gravimetry. (Free water removed before analysis, non-soil objects such as sticks, leaves, grass and stones also removed). US EPA 3550.	0.10 g/100g as rcvd	1-3
Total Recoverable digestion	Nitric / hydrochloric acid digestion. US EPA 200.2.	-	1-3
Total Cyanide Distillation*	Distillation of sample as received. APHA 4500-CN^- C (modified) 23rd ed. 2017.	-	1-3

Table S6- continued.

Sample Type: Sediment			
**Test**	**Method Description**	**Default Detection Limit**	**Sample No**
Total Recoverable Phosphorus	Dried sample, sieved as specified (if required). Nitric/Hydrochloric acid digestion, ICP-MS, screen level. US EPA 200.2.	40 mg/kg dry wt	1-3
Total Recoverable Rubidium	Dried sample, sieved as specified (if required). Nitric/Hydrochloric acid digestion, ICP-MS, trace level. US EPA 200.2.	0.02 mg/kg dry wt	1-3
Total Recoverable Silver	Dried sample, sieved as specified (if required). Nitric/Hydrochloric acid digestion, ICP-MS, trace level. US EPA 200.2.	0.02 mg/kg dry wt	1-3
Total Cyanide*	Distillation, colorimetry. APHA 4500-CN^- C (modified) 23rd ed. 2017 & Skalar Method I295-004(+P14). ISO 14403:2012(E).	0.10 mg/kg dry wt	1-3
Total Nitrogen*	Catalytic Combustion (900°C, O2), separation, Thermal Conductivity Detector [Elementar Analyser].	0.05 g/100g dry wt	1-3
Total Organic Carbon*	Acid pretreatment to remove carbonates present followed by Catalytic Combustion (O2), separation, Thermal Conductivity Detector [Elementar Analyser].	0.05 g/100g dry wt	1-3
Heavy metals, trace As,Cd,Cr,Cu,Ni,Pb,Zn,Hg	Dried sample, <2mm fraction. Nitric/Hydrochloric acid digestion, ICP-MS, trace level.	0.010 - 0.8 mg/kg dry wt	1-3
Acid Herbicides Trace in Soil by LCMSMS*	Solvent extraction, LC-MS/MS analysis. Tested on dried sample. In-house.	0.010 - 0.02 mg/kg dry wt	1-3
Multiresidue Pesticides in Sediment samples by GCMS	Sonication extraction, GC-ECD and GC-MS analysis. In-house based on US EPA 8081 and US EPA 8270.	0.0010 - 0.03 mg/kg dry wt	1-3
Polychlorinated Biphenyls Trace in Soil*	Sonication extraction, GC-MS analysis. In-house based on US EPA 8270.	0.00000020 - 0.035 mg/kg dry wt	1-3
Semivolatile Organic Compounds Trace in Soil by GC-MS	Sonication extraction, GC-MS analysis. Tested on as received sample. In-house based on US EPA 8270.	0.10 - 6 mg/kg dry wt	1-3
7 Grain Sizes Profile as received			
Dry Matter for Grainsize samples (sieved as received)*	Drying for 16 hours at 103°C, gravimetry (Free water removed before analysis).	0.10 g/100g as rcvd	1-3
Fraction >/= 2 mm*	Wet sieving with dispersant, as received, 2.00 mm sieve, gravimetry.	0.1 g/100g dry wt	1-3
Fraction < 2 mm, >/= 1 mm*	Wet sieving using dispersant, as received, 2.00 mm and 1.00 mm sieves, gravimetry (calculation by difference).	0.1 g/100g dry wt	1-3
Fraction < 1 mm, >/= 500 µm*	Wet sieving using dispersant, as received, 1.00 mm and 500 µm sieves, gravimetry (calculation by difference).	0.1 g/100g dry wt	1-3
Fraction < 500 µm, >/= 250 µm*	Wet sieving using dispersant, as received, 500 µm and 250 µm sieves, gravimetry (calculation by difference).	0.1 g/100g dry wt	1-3
Fraction < 250 µm, >/= 125 µm*	Wet sieving using dispersant, as received, 250 µm and 125 µm sieves, gravimetry (calculation by difference).	0.1 g/100g dry wt	1-3
Fraction < 125 µm, >/= 63 µm*	Wet sieving using dispersant, as received, 125 µm and 63 µm sieves, gravimetry (calculation by difference).	0.1 g/100g dry wt	1-3
Fraction < 63 µm*	Wet sieving with dispersant, as received, 63 µm sieve, gravimetry (calculation by difference).	0.1 g/100g dry wt	1-3
Total Petroleum Hydrocarbons in Solids			
Client Chromatogram for TPH by FID	Small peaks associated with QC compounds may be visible in chromatograms with low TPH concentrations. QC peaks are as follows: one peak in the C12 - 14 band, the C21 - 25 band and the C30 - 36 band. All QC peaks are corrected for in the reported TPH concentrations.	-	1
C7 - C9	Solvent extraction, GC-FID analysis. In-house based on US EPA 8015.	20 mg/kg dry wt	1-3
C10 - C14	Solvent extraction, GC-FID analysis. Tested on as received sample. In-house based on US EPA 8015.	20 mg/kg dry wt	1-3
C15 - C36	Solvent extraction, GC-FID analysis. Tested on as received sample. In-house based on US EPA 8015.	40 mg/kg dry wt	1-3
Total hydrocarbons (C7 - C36)	Calculation: Sum of carbon bands from C7 to C36. In-house based on US EPA 8015.	70 mg/kg dry wt	1-3

Table S6- continued.

These samples were collected by yourselves (or your agent) and analysed as received at the laboratory.

Testing was completed between 19-May-2023 and 17-Jul-2023. For completion dates of individual analyses please contact the laboratory.

Samples are held at the laboratory after reporting for a length of time based on the stability of the samples and analytes being tested (considering any preservation used), and the storage space available. Once the storage period is completed, the samples are discarded unless otherwise agreed with the customer. Extended storage times may incur additional charges.

This certificate of analysis must not be reproduced, except in full, without the written consent of the signatory.

Ara Heron BSc (Tech)
Client Services Manager - Environmental

Table S7. Microtox® raw data. Luminescence (l) and gamma (G) measured in the range of concentrations of the three sediment elutriates after 5, 15 and 30 minutes of exposure.

	Site 4679							Site 4680						
**Elutriate %**	I0	I5	I15	I30	G5	G15	G30	I0	I5	I15	I30	G5	G15	G30
0	92	83	76	67	0.9022	0.8261	0.7283	92	94	93	95	1.0220	1.0110	1.0330
0	78	73	67	60	0.9359	0.8590	0.7692	93	85	91	95	0.9140	0.9785	1.0220
0.20	80	67	64	60	0.0973	0.0531	-0.0016	103	93	95	97	0.0719	0.0784	0.0905
0.20	82	68	64	60	0.1082	0.0794	0.0232	103	92	93	95	0.0835	0.1016	0.1135
0.39	83	67	64	60	0.1385	0.0926	0.0357	101	90	93	95	0.0861	0.0802	0.0919
0.39	79	66	62	59	0.1001	0.0735	0.0025	101	89	91	94	0.0983	0.1040	0.1035
0.78	79	66	64	60	0.1001	0.0400	-0.0141	98	92	95	95	0.0309	0.0260	0.0594
0.78	80	66	60	55	0.1140	0.1234	0.0890	100	90	94	94	0.0754	0.0581	0.0926
1.56	80	67	64	60	0.0973	0.0531	-0.0016	99	91	95	96	0.0529	0.0365	0.0591
1.56	77	67	64	60	0.0562	0.0136	-0.0391	103	91	97	95	0.0954	0.0562	0.1135
3.13	78	70	68	63	0.0240	-0.0335	-0.0729	107	98	101	101	0.0567	0.0537	0.0880
3.13	81	70	67	63	0.0634	0.0185	-0.0373	104	96	102	104	0.0485	0.0141	0.0270
6.25	80	70	67	62	0.0503	0.0060	-0.0338	101	97	101	103	0.0077	-0.0053	0.0071
6.25	82	72	70	64	0.0466	-0.0130	-0.0406	108	98	102	105	0.0666	0.0531	0.0564
12.5	80	70	67	61	0.0503	0.0060	-0.0180	102	101	105	109	-0.0225	-0.0337	-0.0389
12.5	81	73	70	66	0.0197	-0.0250	-0.0810	107	103	107	110	0.0054	-0.0053	-0.0009
25	80	72	67	65	0.0211	0.0060	-0.0784	107	105	111	104	-0.0137	-0.0411	0.0566
25	80	72	69	67	0.0211	-0.0231	-0.1060	107	107	111	106	-0.0321	-0.0411	0.0367
50	82	72	72	71	0.0466	-0.0404	-0.1353	108	110	117	122	-0.0497	-0.0818	-0.0908
50	82	72	72	72	0.0466	-0.0404	-0.1473	120	133	142	147	-0.1267	-0.1594	-0.1616

Table S7- continued.

Elutriate %	Site 4681						
	I0	I5	I15	I30	G5	G15	G30
0	92	95	113	127	1.033	1.228	1.38
0	92	97	117	133	1.054	1.272	1.446
0.20	85	97	117	135	-0.0856	-0.0918	-0.1103
0.20	83	97	113	130	-0.1071	-0.0818	-0.0978
0.39	81	95	114	134	-0.1103	-0.1118	-0.1458
0.39	89	102	115	127	-0.0895	-0.0326	-0.0097
0.78	83	94	114	130	-0.0786	-0.0899	-0.0978
0.78	81	95	111	130	-0.1103	-0.0878	-0.1196
1.56	84	97	114	137	-0.0963	-0.0789	-0.1336
1.56	84	100	111	136	-0.1235	-0.054	-0.1272
3.13	91	104	127	143	-0.0869	-0.1043	-0.1008
3.13	90	103	123	143	-0.0882	-0.0853	-0.1107
6.25	89	106	129	145	-0.1239	-0.1376	-0.1327
6.25	91	108	129	148	-0.1208	-0.1182	-0.1312
12.5	88	107	132	152	-0.1418	-0.1667	-0.1819
12.5	87	107	132	155	-0.1516	-0.1761	-0.2069
25	87	111	138	157	-0.1821	-0.212	-0.217
25	90	117	142	163	-0.1973	-0.2077	-0.2198
50	90	121	151	170	-0.2239	-0.255	-0.2519
50	102	128	156	179	-0.1685	-0.1827	-0.1948

Table S8. Physico-chemical parameters of the control and the three sediment elutriate samples at the start of the exposure at the highest tested concentrations for the blue mussel embryo-larval development assay.

	Elutriate (%)	Salinity (psu)	Saturation (%)	DO (mg/L)	pH
**Control**	0	34.2	109	8.8	8.1
**4679**	3.13	n/m	n/m	n/m	7.9
	6.25	n/m	n/m	n/m	7.7
	12.5	n/m	n/m	n/m	7.4
	25	33.1	83	6.6	6.8
	50	31.3	70	5.5	6.5
	100	27.7	37	2.9	6.3
**4680**	25	33.4	106	8.2	7.3
	50	31.8	83	6.7	6.9
	100	28.3	58	4.6	6.5
**4681**	25	32.8	93	7.4	7.6
	50	31.6	86	6.7	7.3
	100	28.1	49	3.9	6.8

Table S9. Blue mussel larvae survival in sediment elutriate samples at a range of concentrations after 48 hours of exposure.

	Survival (%)			
**Elutriate (%)**	**Site 4679**	**Site 4680**	**Site 4681**	**Control**
3.13	47.1	43.7	58.3	59.5
3.13	48.9	40.6	39.4	46.0
3.13	51.0	39.6	58.3	50.0
3.13	51.0	37.6	40.1	45.6
3.13	49.7	62.3	61.1	57.9
6.25	43.4	44.1	57.0	
6.25	50.4	36.3	38.1	
6.25	51.6	41.5	53.9	
6.25	40.8	39.8	45.0	
6.25	40.3	65.4	52.2	
12.5	6.1	21.6	40.4	
12.5	21.1	32.0	34.1	
12.5	5.4	23.6	51.5	
12.5	9.1	29.3	40.7	
12.5	9.5	54.8	67.4	
25	0	0	43.3	
25	0	0	28.0	
25	0	0	46.7	
25	0	0	33.3	
25	0	0	65.0	
50	0	0	34.3	
50	0	0	41.3	
50	0	0	46.4	
50	0	0	23.6	
50	0	0	51.4	
100	0	0	0	
100	0	0	0.8	
100	0	0	0	
100	0	0	0	
100	0	0	0	

Table S10. Concentrations of the detected organic compounds by rapid comprehensive analysis in the three sediment samples collected.

Compound name	CAS no.	Concentration (µg/kg-dry wt)			Source / use
		4679	4680	4681	
Benzyl alcohol	100-51-6	4.4		4.4	Solvent
4-Cymene+	99-87-6		7.1		Solvent
Isophorone+	78-59-1			6.3	Solvent
1-Nonanol	143-08-8			5.4	Solvent, fragrance
3- & 4-Methylphenol+	108-39-4 & 106-44-5	8.4	5.1		Disinfectant, antiseptic, solvent (plastic and resin)
2-Acetyl-5-methylthiophene+	13679-74-8			6.4	Plant, flavouring agent
Benzothiazole	95-16-9			3.9	Vulcaniser, antioxidant, dye intermediate, component of some pesticides and pharmaceuticals
2,6-Dimethylnaphthalene+	581-42-0			111	PAH
1,3-Dimethylnaphthalene+	575-41-7			6.8	PAH
a-Ionone+	127-41-3			16	Plant, fragrance, flavouring agent
b-Ionone+	14901-07-6			48	Plant, fragrance, detergents, flavouring, intermediate in vitamin A, E and K synthesis
2,6-Di-tert-butyl-4-benzoquinone+	719-22-2	31	25		Bacterial product, oxidant, polymerisation catalyst
2-Naphthol	135-19-3	11	17	13	Pigments, fats, oils, insecticides, pharmaceuticals, perfumes, antiseptics, pesticides and antioxidants for rubber, human metabolite (exposure to chlorpyrifos)
Diethyl phthalate	84-66-2			4.1	Plasticiser
Diphenylamine	122-39-4	1.7	0.9	12	Stabiliser, pesticide
4-Nonylphenol+	25154-52-3	258		606	Stabiliser, plasticiser
Tris(1-chloro-2-propyl)phosphate (TCPP)	13674-84-5	20	17	228	Flame retardant
Phenanthrene	85-01-8	5.9	6.9		PAH – coal tar constituent
Diisobutyl phthalate (DIBP)	84-69-5			20	Plasticiser, binding agent

Compound name	CAS no.	Concentration (µg/kg-dry wt)			Source / use
		4679	4680	4681	
Methyl palmitate	112-39-0	33	16	59	Intermediate for detergents, stabilisers, resins, lubricants
Dibutyl phthalate	84-74-2			51	Plasticiser
2-Phenylnaphthalene+	612-94-2			7.1	PAH
Linolelaidic acid methyl ester+	2566-97-4		25		Fatty acid
Methyl stearate	112-61-8	30	21		Lubricant, intermediate for detergents, emulsifiers, stabilisers, plasticisers
Pyrene	129-00-0	8.2	4.3	26	PAH
9-Nitrophenanthrene+	954-46-1	120			PAH
p,p'-DDE	72-55-9	5.5	15.5	24	Pesticide metabolite
o,p'-DDD	53-19-0		5.3	26	Pesticide
p,p'-DDD	72-54-8	5.1	14.1	90	Pesticide
Azamethiphos	35575-96-3	206.4			Pesticide
Diclomezine+	62865-36-5	310.2			Pesticide
Tris(2-ethylhexyl)phosphate	78-42-2	57.7			Flame retardant, plasticiser
Bifenthrin	82657-04-3	36.8			Pesticide
Butyl benzyl phthalate	85-68-7		15.4		Plasticiser
Adipic acid, bis-2-ethylhexylester	103-23-1				Plasticiser, solvent
Benzo(a)anthracene+	56-55-3		6.2		PAH
Bis(2-ethylhexyl)phthalate	117-81-7	111		1029	Plasticiser
Permethrin @1	52645-53-1	148			Pesticide
Permethrin @2	52645-53-1	215			Pesticide
Perylene+	198-55-0	66	47	98	PAH
Coprostanol+	360-68-9	575	73	146	Main sterol of the faeces produced by the reduction of cholesterol by intestinal bacteria
Epicoprostanol+	516-92-7	1307	82	0	Sterol from bile acids and derivatives
Cholesterol+	57-88-5	3240	1324	982	Main sterol of higher animals found in all body tissues. Emulsifier

Compound name	CAS no.	Concentration (µg/kg-dry wt)			Source / use
		4679	4680	4681	
3-Cholestanone	15600-08-5	617		106	Metabolite of cholesterol
Cholestanol+	80-97-7	965	183	420	Steroid ketone derived from coprostanol
Indeno(1,2,3-cd)pyrene	193-39-5			28	PAH
Campesterol+	474-62-4	425	436	540	Phytosterol
Stigmasterol+	83-48-7	751	843	1243	Phytosterol, used in preparation of progesterone and other steroids
24-Ethyl coprostanol+	4736-91-8	302	181	376	Phytosterol
beta-Sitosterol+	83-46-5	2158	2457	2703	Phytosterol, used as emulsifier, stabiliser in cosmetic
Stigmastanol+	83-45-4	455	435	812	Phytosterol, role in anti-cholesterolemic drug
n-C12H26(Dodecane)	112-40-3	7.2			Solvent
n-C14H30(Tetradecane)	629-59-4	35	13	38	Lubricant, stabiliser, solvent, building block for detergents and animal feed
n-C15H32(Pentadecane)	629-62-9	64	9.2	53	Plant oil, solvent, organic synthesis
n-C16H34(Hexadecane)	544-76-3	131	16	116	Plant metabolite, component of gasoline, solvent, organic intermediate
n-C17H36(Heptadecane)	629-78-7	209	29	184	Plant metabolite, paraffin waxes (implies lubricating oil, fuel oil, anticorrosive)
n-C18H38(Octadecane)	593-45-3	180	8.7	147	Plant, bacteria metabolite, paraffin waxes
n-C19H40(Nonadecane)	629-92-5	184	26	176	Plant metabolite, paraffin waxes
n-C20H42(Eicosane)	112-95-8	161	16	144	Plant metabolite, paraffin waxes
n-C21H44(Henicosane)	629-94-7	173	41	174	Plant metabolite, pheromone, paraffin waxes
n-C22H46(Docosane)	629-97-0	129	27	136	Plant metabolite, used in organic synthesis, paraffin waxes
n-C23H48(Tricosane)	638-67-5	127	38	136	Plant metabolite, paraffin waxes
n-C24H50(Tetracosane)	646-31-1	95	18	117	Plant metabolite, paraffin waxes
n-C25H52(Pentacosane)	629-99-2	168	94	189	Plant metabolite, paraffin waxes
n-C26H54(Hexacosane)	630-01-3	74	46	73	Plant metabolite, paraffin waxes
n-C27H56(Heptacosane)	593-49-7	284	172	298	Plant metabolite, paraffin waxes

Compound name	CAS no.	Concentration (µg/kg-dry wt)			Source / use
		4679	4680	4681	
n-C28H58(Octacosane)	630-02-4	119	64	132	Plant metabolite, paraffin waxes
n-C29H60(Nonacosane)	630-03-5	486	433	502	Plant metabolite, paraffin waxes
n-C30H62(Triacontane)	638-68-6		64	121	Natural product (plant, animal), paraffin waxes
n-C31H64(Hentriacontane)	630-04-6	449	471	458	Natural product (plant, animal), paraffin waxes
n-C32H66(Dotriacontane)	544-85-4	109	85	163	Plant, paraffin waxes
n-C33H68(Tritriacontane)	630-05-7	342	304	323	Plant, paraffin waxes

Source: National Center for Biotechnology Information. 2023. PubChem compound summary. [accessed July 2023]. https://pubchem.ncbi.nlm.nih.gov

Figure S1. AIQS-GC screen, AXEL for NAGINATA spectrum for 4-nonylphenol.

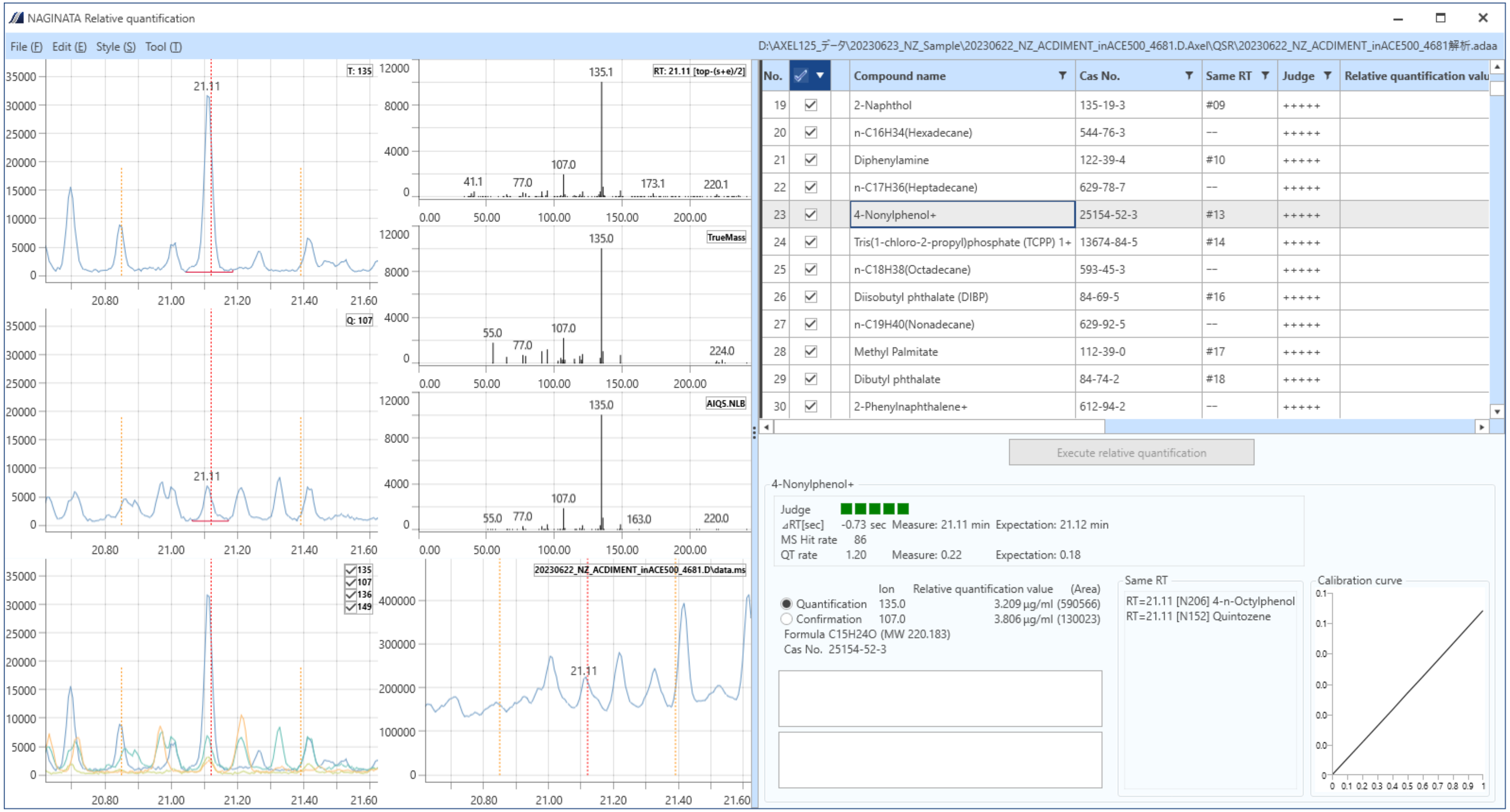

Figure S2. Dose-response of *Aliivibrio fischeri* to an increasing concentration of the reference toxicant.

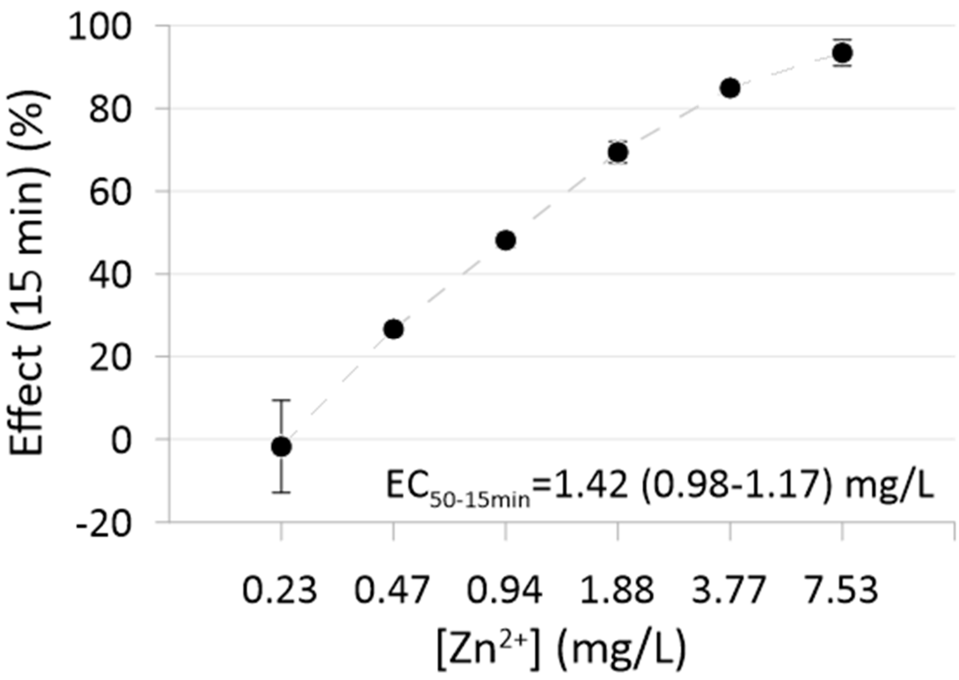

Supplement: Supplementary file 1 [file toxics-12-00558-s001.zip › toxics-3112726-supplementary materials.pdf]
